# Species identity and combinations differ in their overall benefits to *Astragalus adsurgens* plants inoculated with single or multiple endophytic fungi under drought conditions

**DOI:** 10.3389/fpls.2022.933738

**Published:** 2022-09-07

**Authors:** Yi-Ling Zuo, Qian-Nan Hu, Le Qin, Jia-Qiang Liu, Xue-Li He

**Affiliations:** ^1^School of Life Sciences, Hebei University, Baoding, China; ^2^Key Laboratory of Microbial Diversity Research and Application of Hebei Province, Baoding, China

**Keywords:** root endophytes, combination inoculation, promoting effects, soil fungal community, drought stress

## Abstract

Although desert plants often establish multiple simultaneous symbiotic associations with various endophytic fungi in their roots, most studies focus on single fungus inoculation. Therefore, combined inoculation of multiple fungi should be applied to simulate natural habitats with the presence of a local microbiome. Here, a pot experiment was conducted to test the synergistic effects between three extremely arid habitat-adapted root endophytes (*Alternaria chlamydospora, Sarocladium kiliense*, and *Monosporascus* sp.). For that, we compared the effects of single fungus *vs*. combined fungi inoculation, on plant morphology and rhizospheric soil microhabitat of desert plant *Astragalus adsurgens* grown under drought and non-sterile soil conditions. The results indicated that fungal inoculation mainly influenced root biomass of *A. adsurgens*, but did not affect the shoot biomass. Both single fungus and combined inoculation decreased plant height (7–17%), but increased stem branching numbers (13–34%). However, fungal inoculation influenced the root length and surface area depending on their species and combinations, with the greatest benefits occurring on *S. kiliense* inoculation alone and its co-inoculation with *Monosporascus* sp. (109% and 61%; 54% and 42%). Although *A. chlamydospora* and co-inoculations with *S. kiliense* and *Monosporascus* sp. also appeared to promote root growth, these inoculations resulted in obvious soil acidification. Despite no observed root growth promotion, *Monosporascus* sp. associated with its combined inoculations maximally facilitated soil organic carbon accumulation. However, noticeably, combined inoculation of the three species had no significant effects on root length, surface area, and biomass, but promoted rhizospheric fungal diversity and abundance most, with *Sordariomycetes* being the dominant fungal group. This indicates the response of plant growth to fungal inoculation may be different from that of the rhizospheric fungal community. Structural equation modeling also demonstrated that fungal inoculation significantly influenced the interactions among the growth of *A. adsurgens*, soil factors, and rhizospheric fungal groups. Our findings suggest that, based on species-specific and combinatorial effects, endophytic fungi enhanced the plant root growth, altered soil nutrients, and facilitated rhizospheric fungal community, possibly contributing to desert plant performance and ecological adaptability. These results will provide the basis for evaluating the potential application of fungal inoculants for developing sustainable management for desert ecosystems.

## Introduction

Global warming has intensified the appearance of the drought phenomenon, thus aggravating the adverse impact of drought stress, especially in arid desert areas (Zandalinas et al., 2021). As the most important threat to desert ecosystems, drought adversely influences plant growth, including its development and survival (Omer et al., 2020; Terletskaya et al., 2020; Barker et al., 2021). Plants respond to such drought environments both directly and indirectly; furthermore, indirect responses through intimate associations with endophytic fungi have recently received increased attention (Beckers et al., 2017; Trivedi et al., 2020). Therefore, identifying the most beneficial combinations between plant hosts and endophytes may be of particular value for improving the plant growth in desert habitats and mitigating the negative effects on soil ecosystems.

The rhizosphere is known as the soil region surrounding the plant roots, as well as the reservoir of soil microorganisms (Li et al., 2021). Changes in microorganisms significantly influence plant productivity, survival, and stress resistance by acting on geochemical characteristics of subsurface soils, such as material cycling, decomposition rate, and pathogenicity (Bardgett and van der Putten, 2014; Zhang et al., 2020). The rhizospheric fungal community, in particular, plays a central role in improving soil nutrient recycling and availability, resulting in beneficial effects on plant growth and adaptation (Bardgett and van der Putten, 2014). Recent studies have shown that utilizing beneficial endophytic fungi can alter the composition of soil fungal communities and promote the abundance and beneficial interactions of soil fungi (Azcón et al., 2013; Nanjundappa et al., 2019; Li et al., 2020). Furthermore, endophytic fungi could influence plant–rhizosphere interactions to alleviate abiotic stress (Dimkpa et al., 2009). For example, increased production of fungal exopolysaccharides and microbial activity under water-deficit conditions can impact soil water retention and field performance of tomatoes (Le Gall et al., 2021). In this light, optimizing plant–fungal partnership to increase soil microbial abundance and functionality is also critical for the arid desert soil ecosystem.

Fungal endophytes are ubiquitous in the roots of almost all plants, which co-evolved with hosts with their high genetic and functional diversity, endowing plants with diverse resistance and multiple evolutionary strategies (Barnes et al., 2018; Alzarhani et al., 2019; Leroy et al., 2021). Root endophytes isolated from desert habitats have been evidenced to improve plant drought tolerance and performance, enabling the establishment and survival of host plants in a stressful environment (He et al., 2021; Moghaddam et al., 2021). Rodriguez et al. (2008) suggested that the ability to confer drought tolerance to hosts may be a unique genetic resource of endophytic fungi. Under drought stress, endophytic fungi can improve the osmotic adjustment capacity of the host by increasing the production of antioxidant enzymes and active substances, thus reducing water consumption (Xu et al., 2017). Improved nutrition, phytohormones production, and acquired host resistance or immunity induction are also related to endophytic fungi stimulating plant growth and drought tolerance (Mandyam and Jumpponen, 2005; Kour et al., 2019; Fontana et al., 2021). Additionally, fungal endophytes have been found to increase soil fungal abundance and improve microbial community structure, thereby contributing to plant growth and ecological adaptability under water deficit conditions (He et al., 2019). Some beneficial endophytic fungi may even compensate for the low colonization of arbuscular mycorrhizal fungi (AMF) under natural conditions when plant nutrient consumption is challenged (Han et al., 2021). Compared with the well-studied AMF association, fungal endophytes are readily isolated and cultured *in vitro*, which are gradually regarded as promising reciprocal partners in plants (Gonçalves et al., 2021; Zhong et al., 2022).

Most studies have focused on assessing the effects of single strains on plants under drought stress in essentially sterile conditions (González-Teuber et al., 2018; Li et al., 2019a; Hereme et al., 2020). In natural habitats, plant roots are often colonized by multiple fungi species, which together perform complicated ecological functions, rather than a single fungus alone (Durán et al., 2018; Liu et al., 2020a). Additionally, natural habitats are non-sterile environments due to the presence of local microbiota. Therefore, combined inoculation of multiple endophytic fungi simulates natural conditions better than single inoculations, and is thought to be more competitive and effective in a non-sterile natural environment (Baez-Rogelio et al., 2017; Li et al., 2021). Chen et al. (2017) evaluated the effects of combined inoculation of different AMF species on the growth, nutrient absorption, and photosynthesis of cucumber seedlings, which indicated that the AMF composition consists of distant AMF species showing a better synergistic effect than a single or closely related AMF spp. Nanjundappa et al. (2019) reported that under field conditions, the synergistic effect of AMF and *Bacillus* spp. on crop growth and soil nutrient uptake was much greater than that of inoculation with either AMF or *Bacillus* alone. He et al. (2022) suggested that the dual inoculation of dark septate endophytes and *Trichoderma viride* enriched beneficial microbiota, altered soil nutrient status, and might contribute to enhancing the cultivation of medicinal plants in dryland. Hence, it is essential to identify the combinations of different fungal species and determine their subsequent synergistic effects in non-sterile environments, especially with the inclusion of drought stress.

*Astragalus adsurgens* Pall. (Leguminosae) is a native perennial herbaceous mainly distributed in the desert region of Northwest China (Liu et al., 2018). As typical desert herbage and fine forage, this species is a preferred plant for desert ecological restoration and desert grassland planting because it is strong drought and sandstorm resisting plant (Chen et al., 2013). Therefore, considering the concept of sustainability and the need to enhance the growth status and drought resistance of grassland plants, understanding the interaction between plants and beneficial microbes is crucial to receive benefit from the symbiotic mechanisms.

In a previous study, we investigated the influences of several endophytic fungal strains alone from the roots of desert shrubs on the performance of *Hedysarum scoparium* under different soil water conditions with sterile substrates. These strains established a positive symbiosis with the host plant depending on fungal species and water availability (Li et al., 2019b). In the current study, the effects of single and mixed inoculation of three desert endophytic fungi on *A. adsurgens* plants were studied in a greenhouse pot experiment with non-sterile substrates. Since *A. adsurgens* is a desert plant, our pot experiment was conducted under drought stress which simulated the natural conditions for the plant host and the root endophytes. We hypothesize that mixed inoculations of desert endophytic fungi could either promote plant growth or change the rhizospheric fungal community of *A. adsurgens*, and the synergistic effects between mixed inoculations would depend on the combination of different fungal species. Based on our conjecture, we investigated the effects of mixed inoculations on (1) plant biomass (shoot and root biomass), (2) the morphological parameters (plant height, leaf number, root length, surface area, and diameter), (3) soil physicochemical properties, and (4) the rhizospheric fungal community composition. Such data would display endophytic fungal groups that could withstand the drought conditions that impacted host growth, and their potential for improving the stress tolerance and symbiotic performance of *A. adsurgens* in drought-affected arid lands.

## Materials and methods

### Experimental design

A pot experiment was used to carry out single and multiple cross-inoculations of three root endophytic fungi on seedlings of desert plant *A. adsurgens*. Considering the threat of drought in the natural environment, we conducted this pot experiment under drought stress to test the response of the *A. adsurgens* plant to fungal inoculation under conditions of drought stress. The *A. adsurgens* seedlings were inoculated under 8 inoculation treatments, which included a non-inoculated control (C), inoculation with *Alternaria chlamydospora* (A), *Sarocladium kiliense* (S), *Monosporascus* sp. (M), co-inoculation of *A. chlamydospora* and *S. kiliense* (AS), co-inoculation of *A. chlamydospora* and *Monosporascus* sp. (AM), co-inoculation of *S. kiliense* and *Monosporascus* sp. (SM), and the combination of the three species (ASM). There were 5 replicates for each treatment (two plants/pot/replicate), and a total of 40 pots were set up for the experiment. The experiment lasted for 4 months and was performed in a growth chamber with a 14/10 h photoperiod, day/night temperatures of 27/22°C, and 60% mean relative humidity.

### Fungal isolates and plant materials

Endophytic fungal strains of *A. chlamydospora, S. kiliense*, and *Monosporascus* sp. used in this experiment were isolated from the roots of xerophytic shrubs in an extremely arid desert of Northwest China, such as *Sympegma regelii, Salsola passerine*, and *Ephedra przewalskii*. In our previous studies, these three endophytic fungi have been shown to be drought-tolerant *in vitro*, and are therefore considered to confer adaptive benefits to host plants (Zuo et al., 2021, 2022). The identification of the three fungal species was performed based on the similarity alignment of internal transcribed spacer (ITS) sequences in GenBank with the BLASTN program (Supplementary Figure 1). The ITS sequences for the fungi are available in the GenBank database under accession numbers OM304622 (*A. chlamydospora*), OM304621 (*S. kiliense*), and OM304623 (*Monosporascus* sp.). These fungal strains are deposited in the culture collection of the Laboratory of Mycorrhizal Biology, Hebei University, China. All fungal strains were cultured at 27°C in darkness and subcultured every 2 weeks.

Mature seeds of *A. adsurgens* were provided by Linquan Ecological Seed Corporation in Minqin, Gansu Province, China, and stored at 4°C. The seeds were surface-sterilized in a solution of 70% ethanol for 3 min and 2.5% sodium hypochlorite for 10 min with agitation. The sterilized seeds were gently washed several times with sterile water and then aseptically planted onto water–agar medium (containing 10 g/L agar) in petri dishes for germination at 27°C in the dark.

### Inoculation assay and drought stress treatment

Approximately 1,000 g of culture substrate containing 500 g of soil mixed with 500 g of river sand (<2 mm) were placed in sterile plastic pots (11.5 cm height, 13.6 cm diameter at the top, and 9.5 cm diameter at the base) and prepared for inoculation assay. One week old *A. adsurgens* seedlings were transplanted into sterile plastic pots for subsequent growth. The mixture substrates contained 9.98 mg/g of organic matter, 63.85 μg/g of nitrate nitrogen, 94.89 μg/g of ammonia nitrogen, and 16.37 μg/g of available phosphorus. After 1 month of seedlings' acclimation to the pot, fungal endophytes (alone or in combination) were inoculated. The fungal inoculums were produced by sterile culture of fungal strains in Petri dishes using a PDA culture medium and inoculated in the form of liquid suspensions. Specifically, six mycelia discs (9 mm in diameter) from the active mycelium of 10-day-old PDA colonies were transferred to a container of 200 ml sterile water and then were crushed with a homogenizer in intervals of 30 s for 3 min (Del Barrio-Duque et al., 2020). Subsequently, the prepared fungal suspensions were irrigated uniformly in the soil substrates where *A. adsurgens* seedlings grew. The viability of the broken fungal fragments has been confirmed by culturing on a PDA culture medium. Three mycelia discs of each fungus were, respectively, used in the pairwise fungal inoculation treatments, and two discs were, respectively, used in the combination of inoculation with three fungi. For the non-inoculated treatments, the plugs excised from the sterile medium without any fungi were used instead.

One month after inoculation with fungi, pots were kept at low water content (30% field capacity) in accordance with the median value (approximately 28% field capacity) in the natural habitat of *A. adsurgens* in Northwest China to simulate drought stress (Bai et al., 2009). The drought stress experiment lasted for 2 months, and the significant effect of this level of drought has been demonstrated in our previous experiments (Li et al., 2019a,b). Water loss was determined by regular weighing and was supplemented with sterile distilled water to meet the requirements of water capacity.

### Plant biomass and morphological parameters

At the end of the experiment, plant height and stem branching number were recorded for each replicate with two plants in each pot. Shoots and roots from each replicate were separately harvested, and roots were gently washed with deionized water to remove the sand. The root sections for each replicate were floated in water at ~1 cm depth in a plexiglass tray and scanned using a desktop scanner. Morphological characteristics of roots, such as total root length, average root diameter, and root surface area, were determined using the WinRHIZO image analysis system (Chen et al., 2012). The remaining roots and fresh shoots were dried at 80°C for at least 48 h prior to calculating the plant biomass. The total biomass production of plants was the sum of the dry weights of both shoots and roots.

### Soil enzyme activities and physico-chemical properties

Rhizospheric soils from each replicate were collected when harvesting plants. Part of the soil samples was stored at 4°C for enzyme analyses, the subsamples were air-dried at room temperature to determine soil physicochemical properties, while the remaining soils were frozen at −80°C for further analysis of fungal community composition. Soil pH was determined with a digital pH meter in a (1:2.5, soil: water) suspension. The activities of soil urease (U) and acid phosphatase were determined, respectively, using the method of Hoffmann and Teicher (1961) and Tarafdar and Marschner (1994). Soil organic carbon (SOC) was estimated according to the potassium dichromate oxidation method (Rowell, 1994). Soil nitrate and ammonia were examined by a continuous flow analyzer (Smart Chem 200, Alliance, France) *via* extraction in a 2 mol/L of KCl solution (1:5 w/v), during which samples were shaken for 1 h at 250 r min^−1^ and then filtered. Soil available phosphorus (P) was extracted for 30 min by using 0.5 M sodium bicarbonate and examined by the chlorostannus-reduced molybdophosphoric blue color method (Olsen et al., 1954).

### Soil fungal community composition

The genomic DNA of soil samples was extracted using a Powersoil^®^ DNA Extraction kit. The DNA concentration and purity were determined using a NanoDrop 2000 UV-vis spectrophotometer, and DNA quality was checked with 1% agarose gel electrophoresis. The universal primers of ITS1F (5′-CTTGGTCATTTAGGAAGTAA-3′) and ITS2R (5′-GCTGCGTTCTTCATCATGATGC-3′) were employed to generate the amplification of fungal internal transcribed spacer 1 (ITS1) regions with a thermocycler PCR system (GeneAmp 9700, Applied Biosystems ABI, Foster City, CA, USA). The PCR reactions were performed in 20 μl mixtures containing 4 μl of 5 × FastPfu Buffer, 2 μl of 2.5 mM dNTPs, 0.8 μl of each primer (5 μM), 0.4 μl of FastPfu Polymerase, and 10 ng of template DNA. The PCRs were conducted using the following program: 3 min of denaturation at 95°C, 27 cycles of 30 s at 95°C, 30 s for annealing at 55°C, 45 s for elongation at 72°C, and a final extension at 72°C for 10 min. The resulting PCR products were extracted from a 2% agarose gel and further purified using the AxyPrep DNA Gel Extraction Kit (Axygen Biosciences, Union City, CA, USA) and quantified using a QuantiFluorTM -ST system (Promega, Madison, WI, USA) according to the manufacturer's protocol. The purified amplicons were pooled in equimolar amounts and paired-end sequenced [2 × 300 base pairs (bp)] using the Illumina MiSeq platform (PE300, Illumina, San Diego, CA, USA) with standard protocols by Majorbio Bio-Pharm Technology Co., Ltd. (Shanghai, China).

The Raw FASTQ sequences were quality-filtered using Trimmomatic software and merged using FLASH software to obtain valid and high-quality sequences. All reads were assembled according to their overlapping sequences longer than 10 bp, and sequences that could not be assembled were discarded. The maximum mismatch ratio of overlapping regions was 0.2. Samples were distinguished according to the barcodes. Operational taxonomic units (OTUs) were clustered by UPARSE (version 7.0.1090, http://www.drive5.com/uparse/) according to 97% similarity with a novel “greedy” algorithm that performs chimera filtering and OTU clustering simultaneously after dereplication and discarding all singletons (Edgar, 2013). The taxonomy of each representative sequence was analyzed with the RDP Classifier algorithm (http://rdp.cme.msu.edu/) against the UNITE version 8.0 database (https://unite.ut.ee/) using a confidence threshold of 70% (Kõljalg et al., 2013). To eliminate the effects of the different numbers of sequences among the samples on the identified fungal community, the number of sequences per sample was normalized to the smallest sample size using the subsample command in MOTHUR. Subsequently, rarefaction curves were assembled, and the alpha diversity indices of OUT richness, Simpson index, Shannon diversity, and evenness were calculated. The relative abundance of specific fungal taxa was defined as the number of reads of a particular taxon, which is a percentage of the number of all reads in a sample.

### Statistical analysis

The Shapiro–Wilk test and Bartlett's test were employed to check the normality and homoscedasticity of all the data, respectively. One-way analysis of variance (ANOVA) was applied for plant growth and rhizospheric soil parameters among treatments, and Duncan's multiple-range tests were performed (*p* < 0.05). For the sequenced rhizospheric fungal community, comparisons of the fungal alpha diversity were examined by Student's tests for paired comparisons between samples (De Winter, 2013). A Venn diagram was used to visualize the numbers of fungal OTUs that were shared among treatments. Non-metric multidimensional scaling (NMDS) was used to visualize the community composition dissimilarities of soil fungi based on the Bray–Curtis, and the analysis of similarity (ANOSIM) was used to examine significant differences based on 999 permutations (Clarke et al., 2006). The relative abundances of the top 50 abundant fungal OTUs were depicted using the “pheatmap” function to display the clustering of fungal community composition among different treatment groups. Linear discriminant analysis (LDA) effect size (LEfSe), which can take into account the statistical significance and biological relevance, was conducted to identify OTUs differentially represented between different inoculation treatments by using the non-parametric factorial Kruskal–Wallis (KW) sum-rank test and LDA (Segata et al., 2011; Guerrero-Preston et al., 2016). High LDA scores reflect a significantly higher abundance of certain taxa. The fungal groups that were significantly correlated with soil factors and plant growth parameters were identified based on the coefficient of Pearson's correlation, and the *p*-value was adjusted based on the false discovery rate (FDR) (Yao et al., 2019; He et al., 2022). The interrelationships among soil factors, the fungal community, and plant growth parameters were evaluated by analysis of Pearson's correlation and structural equation modeling (SEM) *via* AMOS 21.0. The SEM procedure was started with an a priori model on the basis of our predictions and related literature. The chi-square (χ^2^) test, *p*-value, comparative fit index (CFI), and root mean square error of approximation (RMSEA) were applied to assess model fit. All the statistical analysis and plotting of graphs were performed by SPSS 19.0 and R package vegan.

## Results

### Plant shoot and root biomass

**Figure 1:**
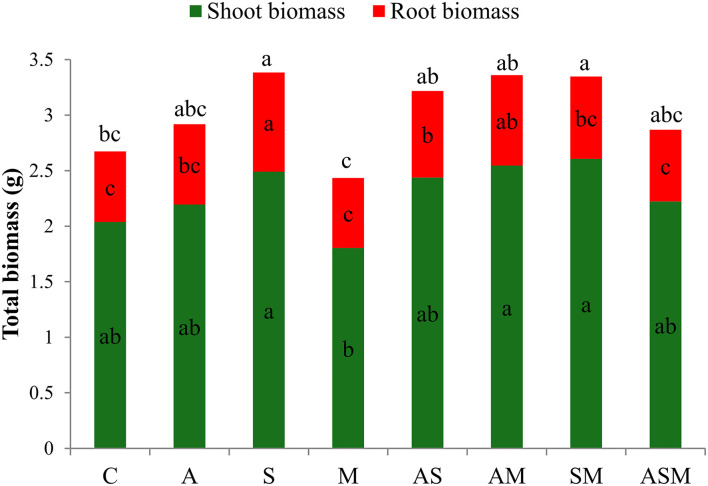
Effects of combined inoculation effects of root endophytic fungi on plant biomass of *A. adsurgens* seedlings. Different lowercase letters indicate significant differences (*p* < 0.05). C, non-inoculated control; A, inoculation with *Alternaria chlamydospora*; S, inoculation with *Sarocladium kiliense*; M, inoculation with *Monosporascus* sp.; AS, co-inoculation of *Alternaria chlamydospora* and *Sarocladium kiliense*; AM, co-inoculation of *Alternaria chlamydospora* and *Monosporascus* sp.; SM, co-inoculation of *Sarocladium kiliense* and *Monosporascus* sp.; ASM, combination inoculation of the three species.

Despite the drought stress imposed, all inoculated seedlings were green, alive, and healthy after 4 months of growth, including the control seedlings. Compared with the non-inoculated plants, fungal inoculation did not influence the shoot biomass of *A. adsurgens* seedlings but obviously influence the root biomass (Figure 1). Under the treatment of single fungus inoculation, the root biomass showed the maximum value when inoculated with S alone. However, in terms of 2-species inoculations, the shoot biomass under AM and SM were higher than that of M inoculated alone, and inoculated with AS and AM induced an increase in root biomass than that of M and non-inoculated plants. Overall, the total biomass of *A. adsurgens* plants under S alone and SM inoculations were significantly higher than that of control plants.

### Plant growth and root morphology traits

**Figure 2:**
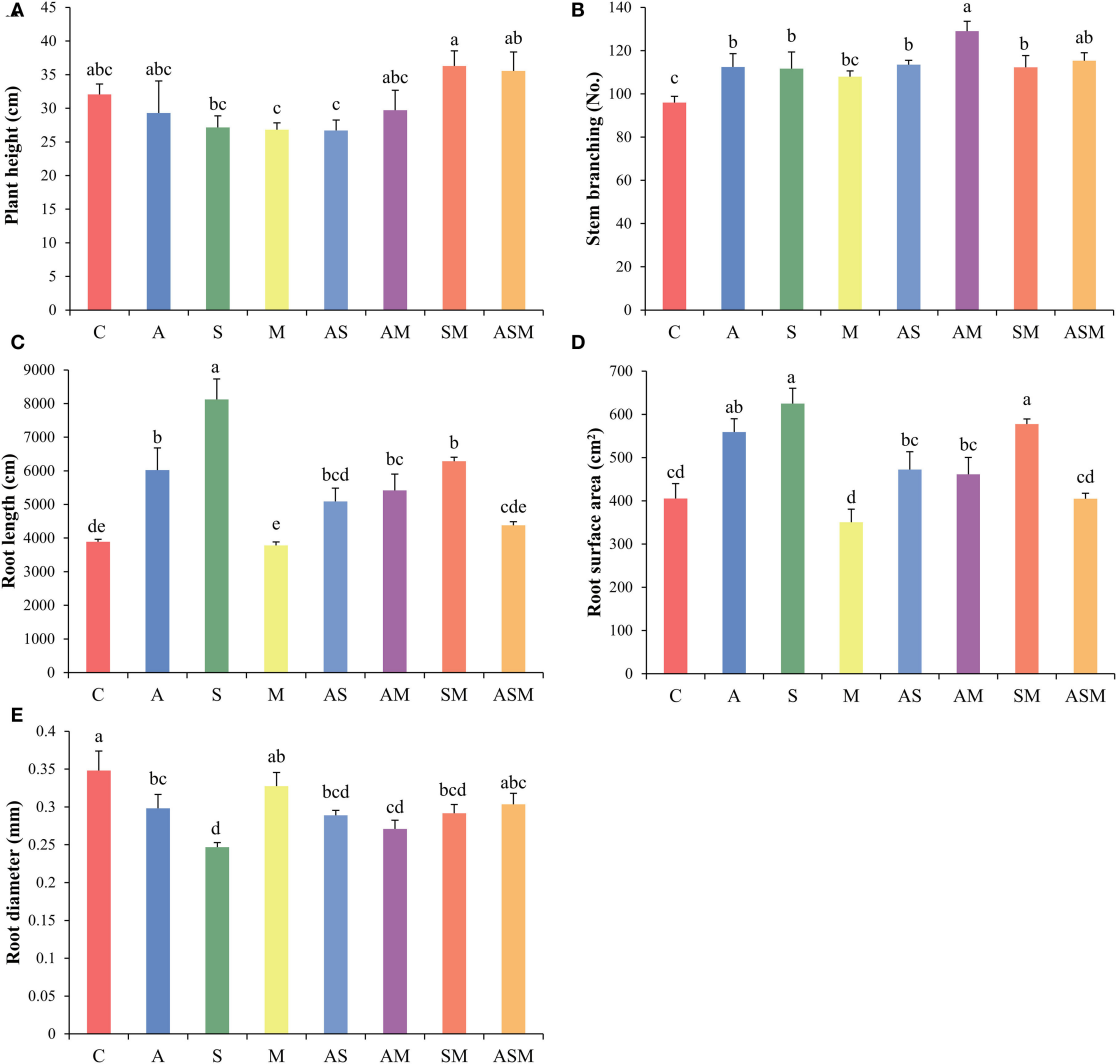
Effects of combined inoculation effects of root endophytic fungi on the morphological parameters of *A. adsurgens* seedlings. **(A)** Plant height, **(B)** stem branching, **(C)** root length, **(D)** root surface area, and **(E)** root diameter. Different lowercase letters indicate significant differences (*p* < 0.05). C, non-inoculated control; A, inoculation with *Alternaria chlamydospora*; S, inoculation with *Sarocladium kiliense*; M, inoculation with *Monosporascus sp*.; AS, co-inoculation of Alternaria *chlamydospora* and *Sarocladium kiliense*; AM, co-inoculation of Alternaria *chlamydospora* and *Monosporascus sp*.; SM, co-inoculation of *Sarocladium kiliense* and *Monosporascus sp*.; ASM, combination inoculation of the three species.

Fungal combination inoculation influenced the growth of *A. adsurgens* seedlings (Figure 2). Regardless of the single or combined inoculation, all fungal inoculation decreased the plant height of *A. adsurgens* seedlings, but the 2-species inoculation of SM and 3-species inoculation of ASM slightly promoted plant height growth (Figure 2A). Compared with the control plant, all fungal inoculation increased the stem branching numbers of *A. adsurgens* except M inoculation; and the AM inoculation had the greatest effect on plant height, which was higher than that of A and M inoculation alone (Figure 2B).

Inoculated with A and S alone increased the total root length and root surface area of *A. adsurgens* to the greatest extent, whereas AS showed a lower promotion effect than every single inoculation compared with the control treatment (Figures 2C,D). The inoculation of M showed no significant difference with the control plant, but the 2-species inoculations of AM and SM existed promoting effects on the root growth of *A. adsurgens* seedlings. However, there was no difference between inoculated *A. adsurgens* and control plants when the three fungal species were combined inoculated. In contrast, all fungal inoculation had a negative impact on root diameter, except M and the 3-species inoculation of ASM (Figure 2E).

### Soil enzymes and physicochemical properties

**Figure 3:**
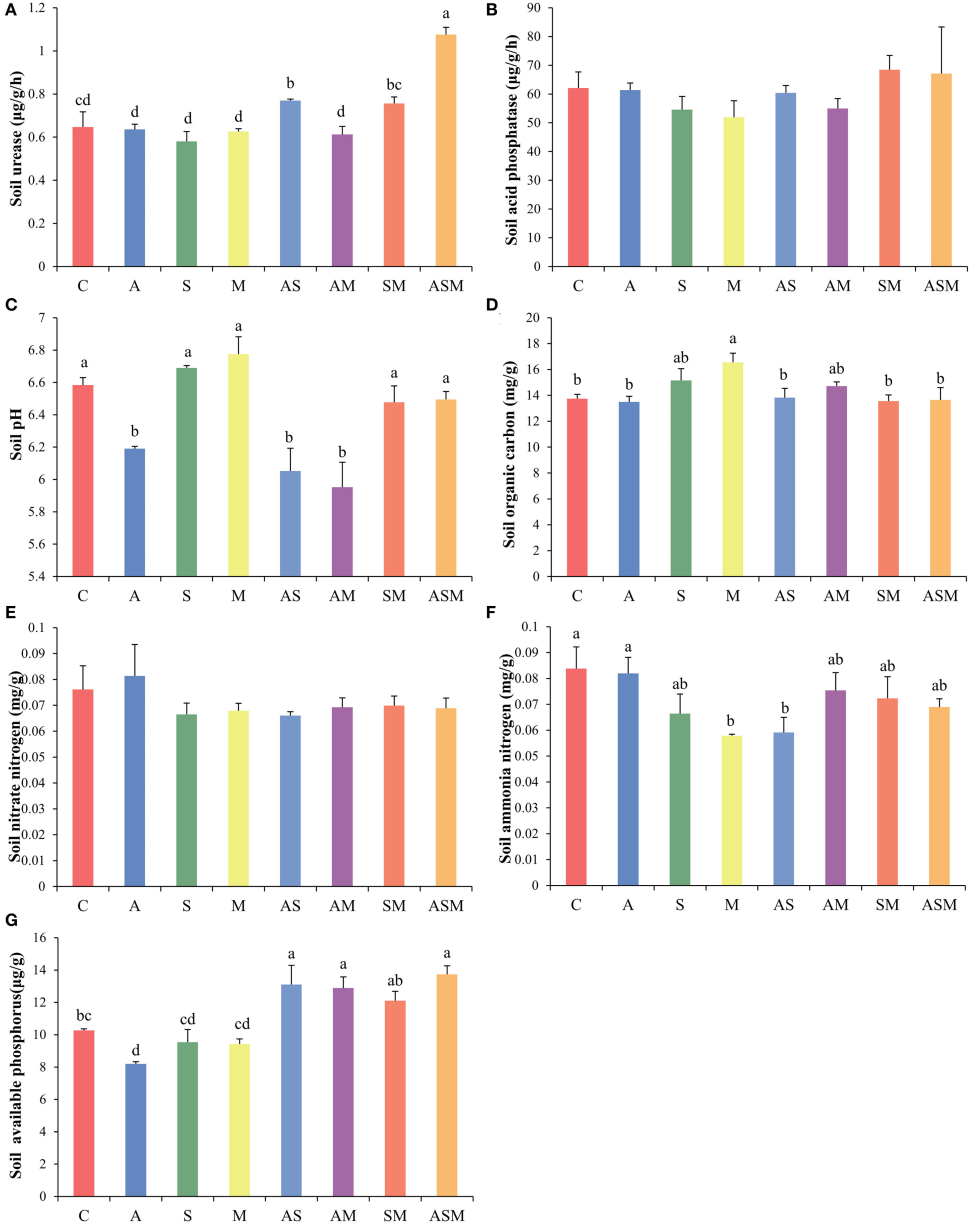
Effects of combined inoculation effects of root endophytic fungi on soil enzymes and physicochemical properties of *A. adsurgens* seedlings. **(A)** Soil urease, **(B)** soil acid phosphatase, **(C)** soil pH, **(D)** soil organic carbon, **(E)** soil nitrate, **(F)** soil ammonia, and **(G)** soil available phosphorus. Different lowercase letters indicate significant differences (p < 0.05). C, non-inoculated control; A, inoculation with Alternaria *chlamydospora*; S, inoculation with *Sarocladium kiliense*; M, inoculation with *Monosporascus sp*.; AS, co-inoculation of *Alternaria chlamydospora* and *Sarocladium kiliense*; AM, co-inoculation of *Alternaria chlamydospora* and *Monosporascus sp*.; SM, co-inoculation of *Sarocladium kiliense* and *Monosporascus sp*.; ASM, combination inoculation of the three species.

The activity of soil urease and acid phosphatase was not influenced by fungal inoculations, but only inoculations with AS and ASM were associated with higher soil urease activity (Figures 3A,B). In terms of soil physicochemical properties, inoculations of A, AS, and AM all resulted in a decrease in soil pH (Figure 3C). Although there was no effect on the morphological growth of *A. adsurgens* seedlings, M inoculation alone increased soil organic carbon content to the greatest extent (Figure 3D). Fungal inoculation treatments did not have an effect on soil nitrate and seemed to reduce the content of soil ammonia, with the remarkably reduced soil ammonia under M and AS inoculations (Figures 3E,F). Compared with the control treatment, inoculation with A, S, and M alone resulted in an insignificant decrease in soil available phosphorus, but 2-species inoculations of AS, AM, SM, and 3-species inoculation of ASM significantly increased the availability of soil available phosphorus (Figure 3G).

### Soil fungal community composition

**Figure 4:**
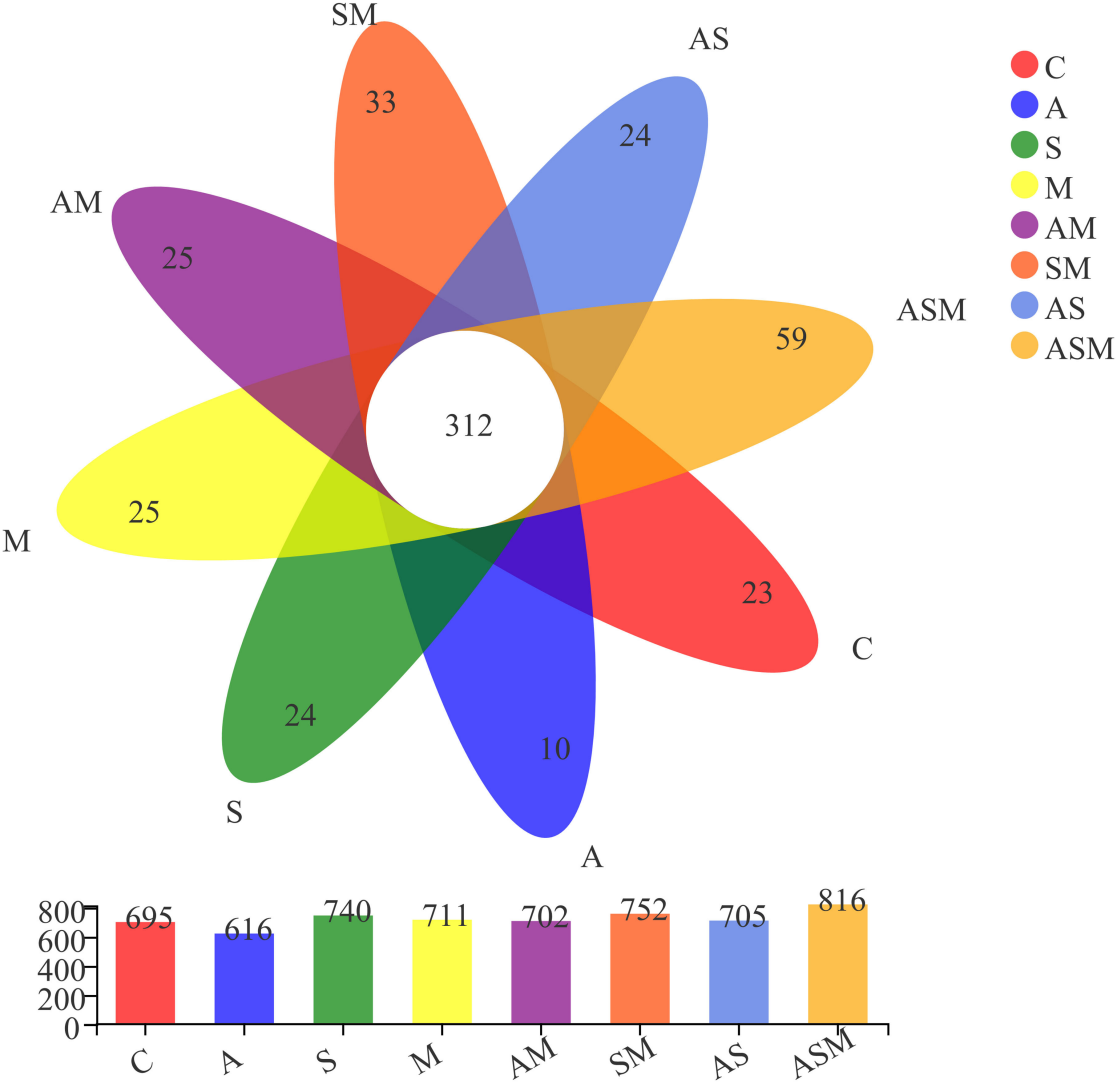
Venn diagram displaying the overlap in fungal OUTs composition of *A. adsurgens* under different fungal inoculation treatments. C, non-inoculated control; A, inoculation with *Alternaria chlamydospora*; S, inoculation with *Sarocladium kiliense*; M, inoculation with *Monosporascus* sp.; AS, co-inoculation of *Alternaria chlamydospora* and *Sarocladium kiliense*; AM, co-inoculation of *Alternaria chlamydospora* and *Monosporascus* sp.; SM, co-inoculation of *Sarocladium kiliense* and *Monosporascus* sp.; ASM, combination inoculation of the three species.

A total of 1,183,752 non-chimeric effective fungal sequences were obtained after removing sequences of low quality, potential chimeras, and singletons. Subsequently, the number of sequences per sample was normalized to the smallest sample size (49,323 reads), and the remaining effective fungal sequences were finally clustered into 1,275 operational taxonomic units (OTUs) at 97% sequence similarity level. The identified fungal OTUs were dominated by *Ascomycota* (94.36%) and represented by 21 classes, 68 orders, 176 families, 396 genera, and 689 species. Of 1,275 fungal OTUs, 312 were shared by all the fungal inoculation treatments (Figure 4). The inoculation of ASM represented the highest number of unique OTUs (59), while the lowest number of unique OTUs (10) occurred under the A inoculation alone. There were 23, 24, 25, 24, 25, and 33 OTUs present only in the non-inoculated control, A inoculation, M inoculation, and 2-species inoculations of AS, AM, and SM, respectively. Rarefaction curves for the Sobs index at the OTU level across all samples approached an asymptote and showed that the overall fungal diversity was well represented (Supplementary Figure 2).

**Figure 5:**
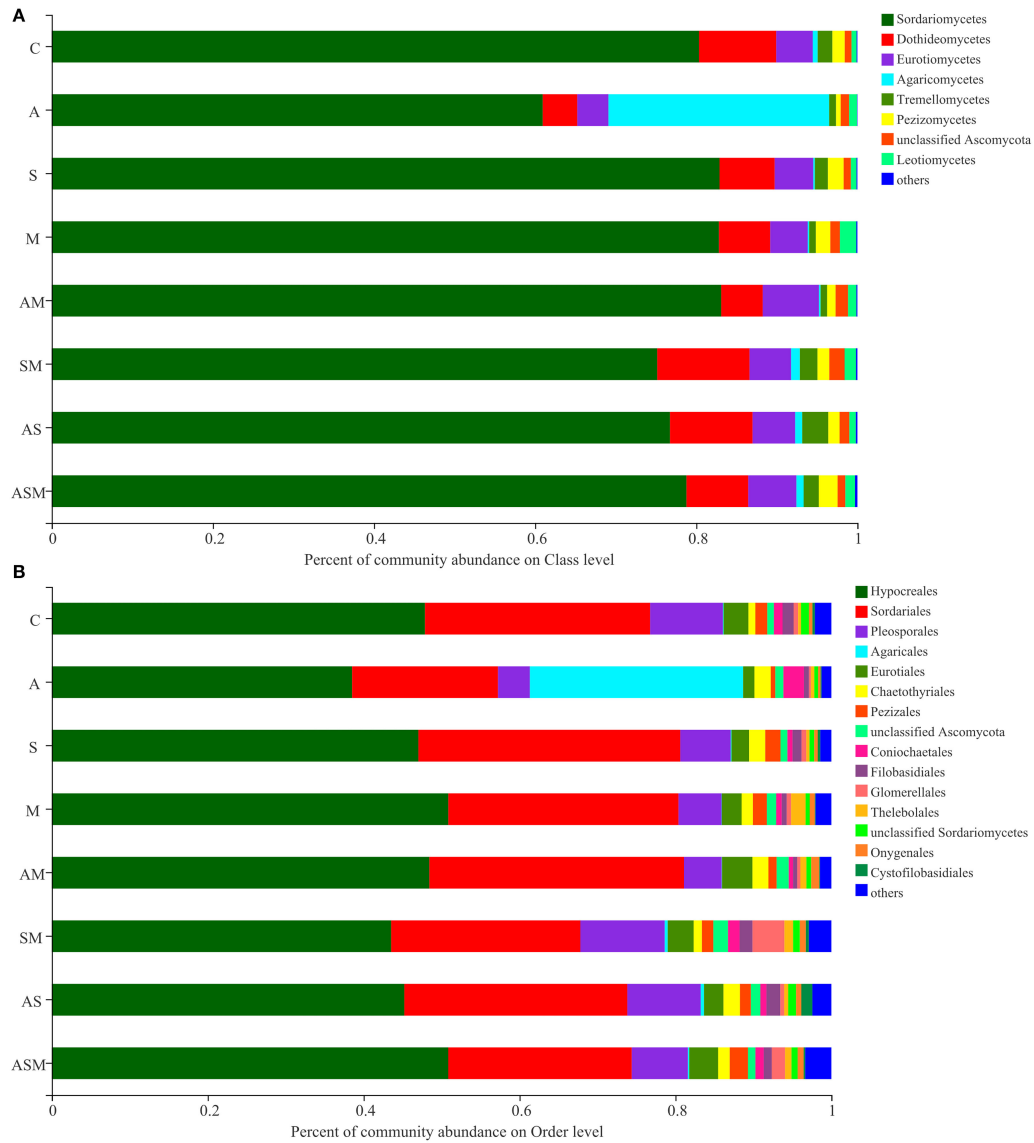
Relative abundances of soil fungi on class **(A)** and order **(B)** levels of *A. adsurgens*. The fungal class and order represents <0.01% of the total reads and were all assigned to “Others.” C, non-inoculated control; A, inoculation with *Alternaria chlamydospora*; S, inoculation with *Sarocladium kiliense*; M, inoculation with *Monosporascus* sp.; AS, co-inoculation of *Alternaria chlamydospora* and *Sarocladium kiliense*; AM, co-inoculation of *Alternaria chlamydospora* and *Monosporascus* sp.; SM, co-inoculation of *Sarocladium kiliense* and *Monosporascus* sp.; ASM, combination inoculation of the three species.

The member of *Sordariomycetes* was the dominant class, with a relative abundance range of 60.94–83.10% across the various treatments (Figure 5A). However, the member of *Agaricomycetes* (27.40%) was the most abundant under A inoculated alone when compared with other inoculation treatments. At the level of order classification, *Hypocreales* and *Sordariales* were the dominant fungal taxon, with relative abundance ranging between 38.53–50.87% and 18.72–33.60%, respectively (Figure 5B). The members of *Agaricales* (27.36 %) and *Coniochaetales* (2.63 %) were mainly distributed under A inoculated alone, while a member of *Glomerellales* (4.09%) was mainly distributed in the inoculation of SM.

### Soil fungal alpha and beta diversities

**Figure 6:**
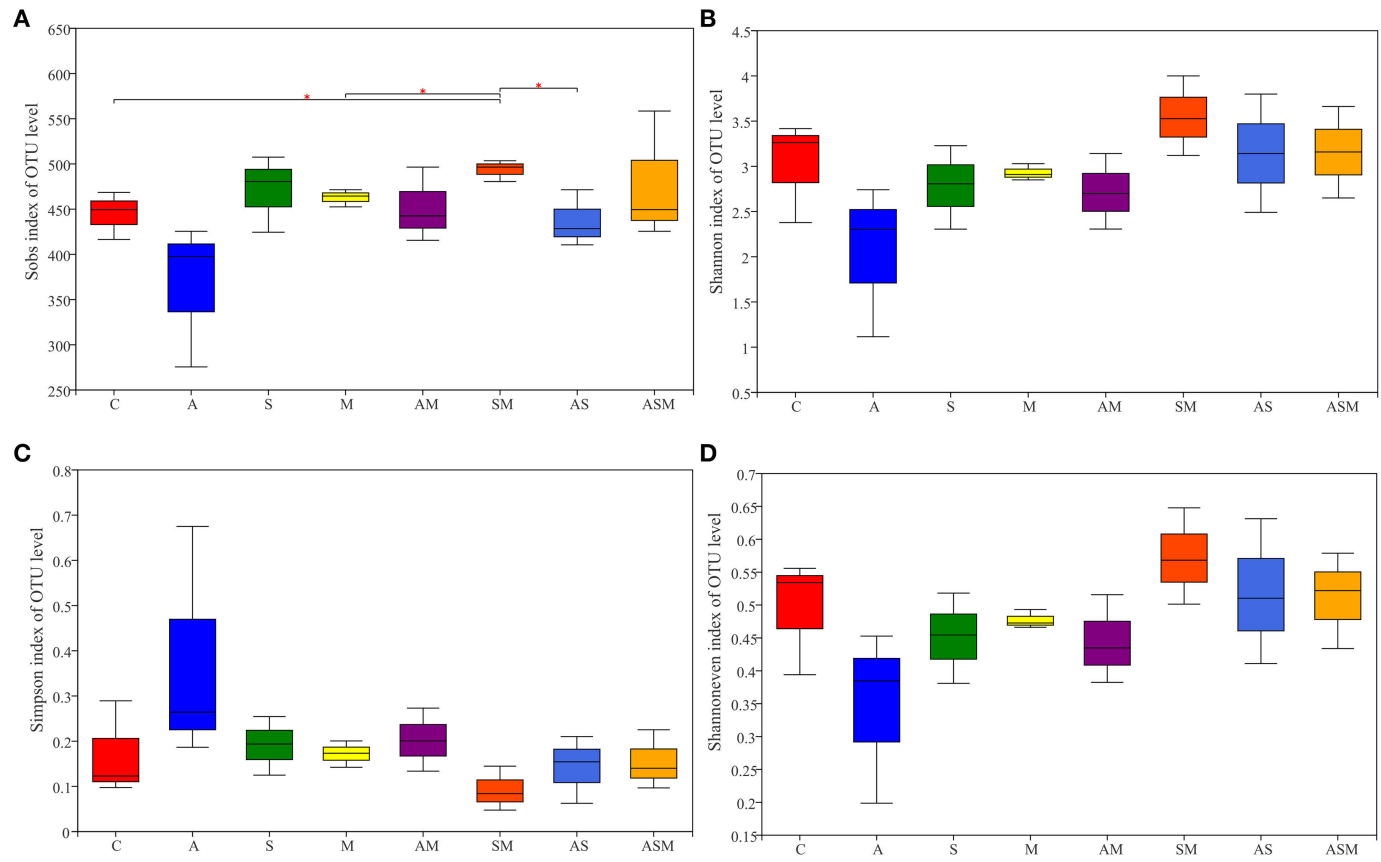
Species richness and diversity index of soil fungi of *A. adsurgens*. **(A)** OTU richness estimated by sobs index; **(B)** Shannon diversity; **(C)** Simpson diversity; **(D)** Shannon's evenness. C, non-inoculated control; A, inoculation with *Alternaria chlamydospora*; S, inoculation with *Sarocladium kiliense*; M, inoculation with *Monosporascus* sp.; AS, co-inoculation of *Alternaria chlamydospora* and *Sarocladium kiliense*; AM, co-inoculation of *Alternaria chlamydospora* and *Monosporascus* sp.; SM, co-inoculation of *Sarocladium kiliense* and *Monosporascus* sp.; ASM, combination inoculation of the three species.

Sobs index values were calculated to describe the observed OTU richness. Alpha diversity, including the Simpson index, Shannon diversity, and evenness index, were analyzed based on OTU richness (Figure 6). The OTU richness of soil fungi under the inoculation of SM was significantly higher than that in non-inoculated control, and inoculations of M and AS (Figure 6A). Inoculation with A alone revealed the lowest soil fungal diversity, while SM slightly promoted soil fungal diversity (Figures 6B,C). The inoculation of A alone also caused a decrease in the evenness of the soil fungal community (Figure 6D).

Nonmetric multidimensional scaling (NMDS) showed that inoculation treatment had no significant effect on the composition of the soil fungal community (Supplementary Figure 3). However, the clustering heatmap based on the top 50 most abundant fungal OTUs indicated that the abundance of soil fungi under different inoculation treatments changed. Moreover, soil fungal composition associated with A and SM inoculations was far away from other inoculation treatments and revealed certain differences (Supplementary Figure 4).

### Linear discriminant analysis of effect size

**Figure 7:**
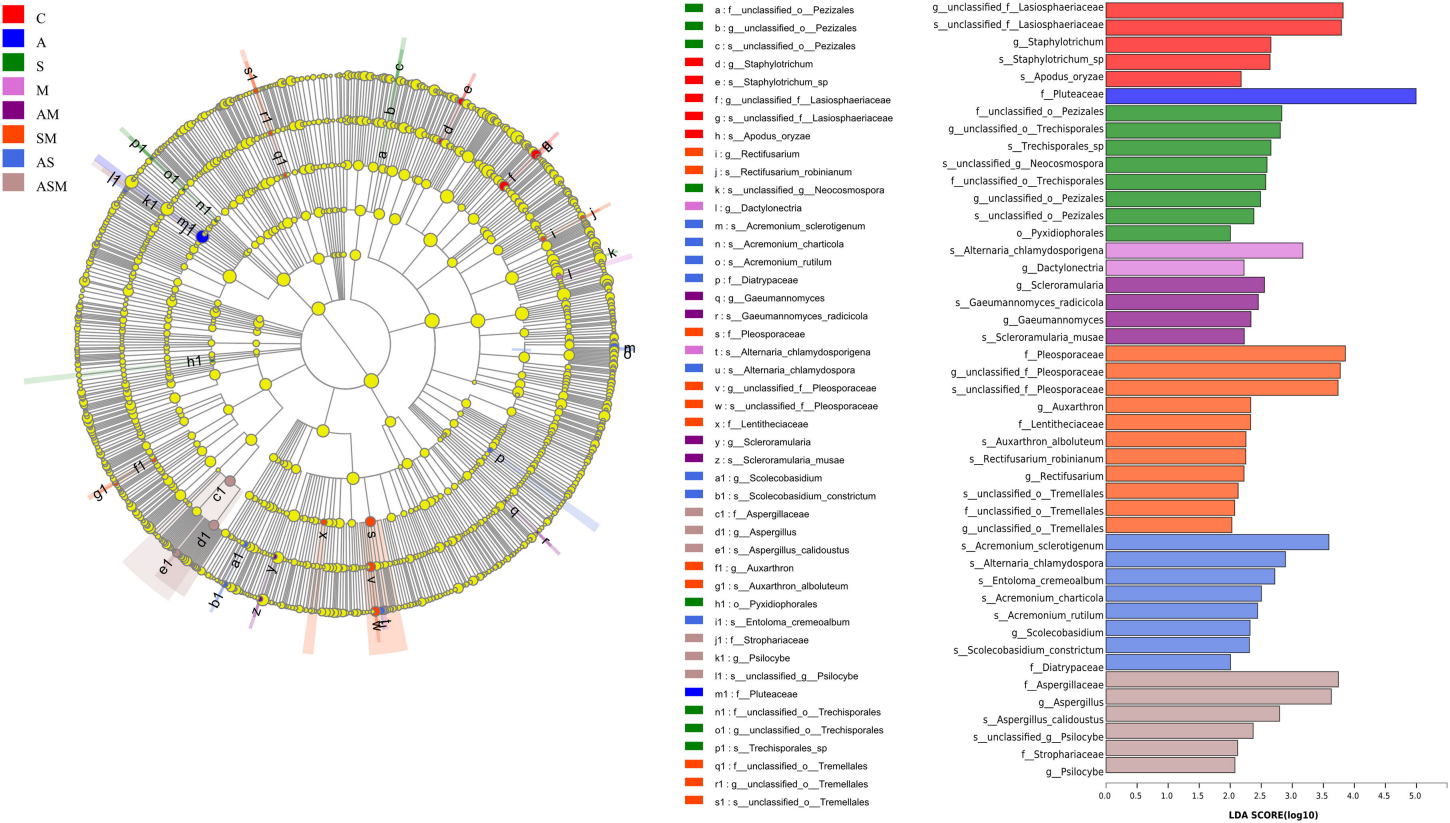
Indicator fungal species with LDA scores of 2 or greater in the rhizosphere of *A. adsurgens* under different inoculation treatments. LDA Effect Size (LEfSe) algorithm was used on OTUs level to determine taxa that were differentially represented among inoculation treatments. C, non-inoculated control; A, inoculation with *Alternaria chlamydospora*; S, inoculation with *Sarocladium kiliense*; M, inoculation with *Monosporascus* sp.; AS, co-inoculation of *Alternaria chlamydospora* and *Sarocladium kiliense*; AM, co-inoculation of *Alternaria chlamydospora* and *Monosporascus* sp.; SM, co-inoculation of *Sarocladium kiliense* and *Monosporascus* sp.; ASM, combination inoculation of the three species.

We used linear discriminant analysis (LDA) of effect size (LEfSe) to determine the taxa that most likely explain the differences of soil fungi among inoculation treatments. Indicator fungal species with LDA scores of 2 or greater in the fungal community were confirmed to predict the best biomarker for each category (Figure 7). The significant inoculation effect observed among different treatment levels was explained by 5, 1, 8, 2, 4, 11, 8, and 6 indicator fungi species, respectively. For example, the taxon of *Pluteaceae* was significantly enriched under A inoculation alone; *Scleroramularia, Gaeumannomyces radicicola, Gaeumannomyces, Scleroramularia musae*, and an unclassified *Pleosporaceae* were significantly enriched under the inoculation of AM; and two unclassified *Pleosporaceae* and *Auxarthron* were significantly enriched under the inoculation of SM. The inoculation of SM presented the most abundant species with significantly different effects (11 indicator taxa).

### Fungal taxa associated with soil factors and plant growth

**Figure 8:**
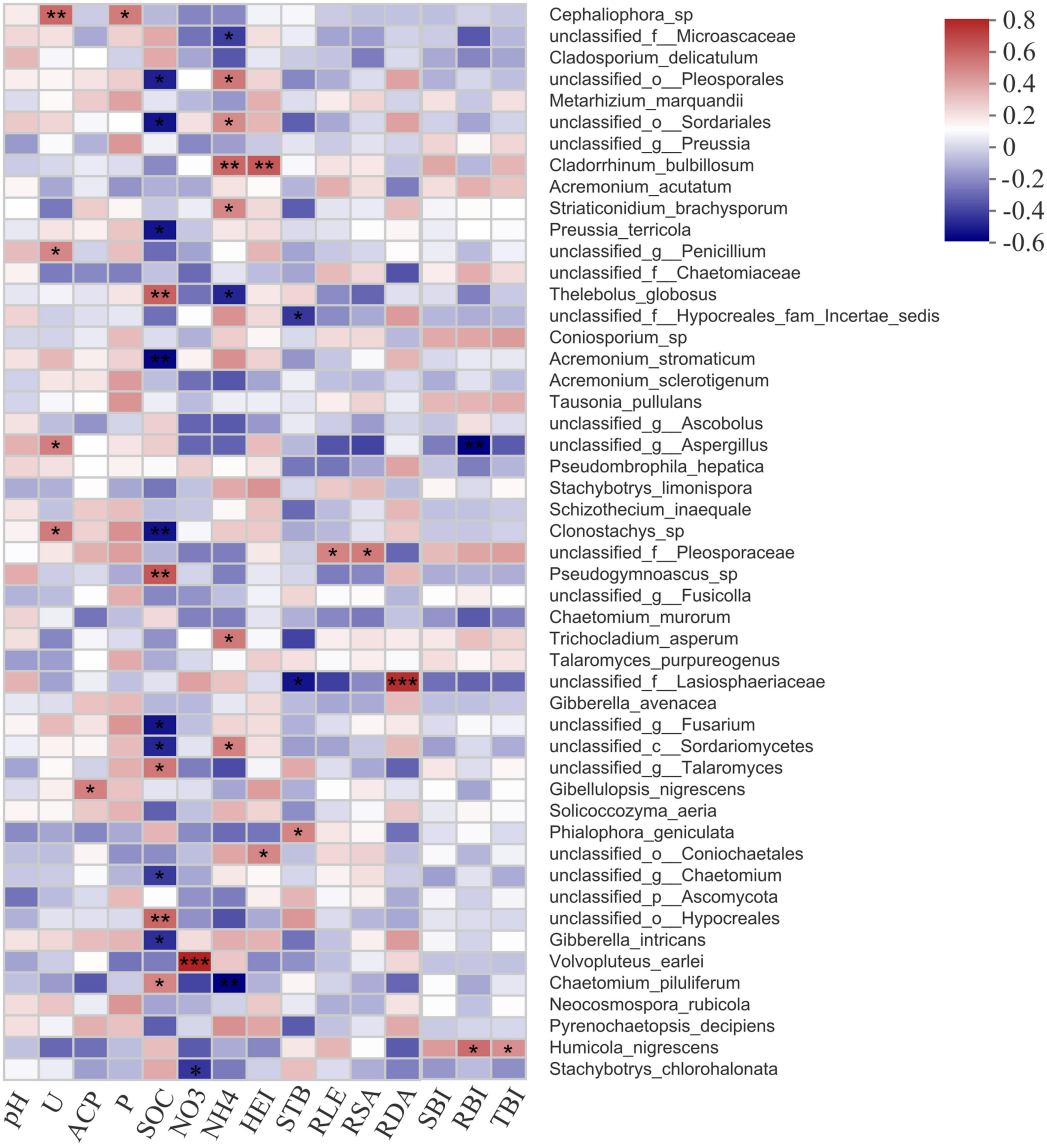
Correlation analysis results showing fungal species associated with soil physicochemical properties and plant growth parameters. U, soil urease; ACP, soil acid phosphatase; P, soil available phosphorus; SOC, soil organic carbon; NO3, soil nitrate; NH4, soil ammonia; HEI, plant height; STB, stem branching numbers; RLE, root length; RSA, root surface area; RDA, root diameter; SBI, shoot biomass; RBI, root biomass; TBI, total biomass. Significance level is shown at: *p < 0.05; **p < 0.01; ***p < 0.001.

The relationship between fungal taxa with the abundance of top 50 and soil factors, as well as plant growth parameters, was presented in heatmap (Figure 8). Members of *Cephaliophora* sp., unclassified *Penicillium*, unclassified *Aspergillus*, and *Clonostachys* sp. were highly correlated with soil urease; *Gibellulopsis nigrescens* and *Cephaliophora* sp. had a positive effect on soil acid phosphatase and available phosphorus, and *Volvopluteus earlei* and *Stachybotrys chlorohalonata* were related to soil nitrate. However, most fungal species revealed a close relationship with soil organic carbon (14 species) and soil ammonia (9 species), such as unclassified *Sordariomycetes, Chaetomium piluliferum*, unclassified *Pleosporales*, and unclassified *Sordariales*.

In terms of plant growth parameters, *Cladorrhinum bulbillosum*, unclassified *Coniochaetales*, unclassified *Hypocreales fam Incertae sedis*, unclassified *Lasiosphaeriaceae*, and *Phialophora geniculate* highly corresponded to plant height and stem branching numbers. Several fungal species were significantly correlated with the root growth and biomass, for example, unclassified *Pleosporaceae*, as well as unclassified *Lasiosphaeriaceae*, were corresponded to root length, surface area, and diameter, and unclassified *Aspergillus* and *Humicola nigrescens* were correlated with root biomass.

### Interrelationship among soil factors, fungi community and plant growth

**Figure 9:**
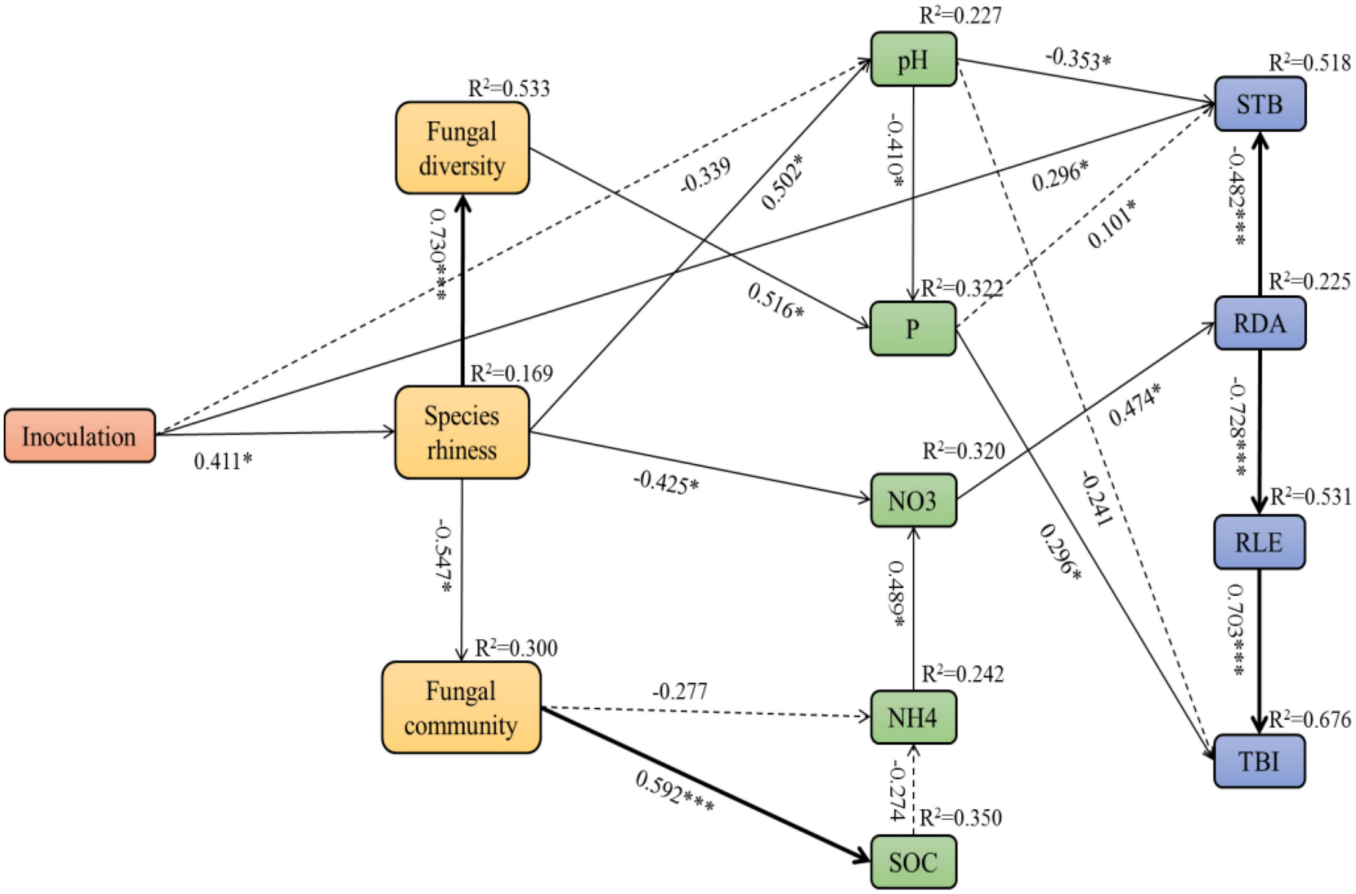
Structural equation model (SEM) showing the causal relationships among soil fungal community, soil factors, and plant growth parameters of *A. adsurgens* associated with different inoculation treatments. The solid lines and dashed lines indicate significant and non-significant pathways, respectively. The width of the solid lines indicates the strength of the causal effect, and the numbers near the arrows indicate the standardized path coefficients. Significant values are indicated by *(*p* < 0.05), **(*p* < 0.01), and ***(*p* < 0.001). The numbers in the upper-right corner of the box indicate the *R*^2^ values and represent the proportion of variance explained for each variable. P, soil available phosphorus; SOC, soil organic carbon; NO_3_, soil nitrate; NH_4_, soil ammonia; STB, stem branching numbers; RLE, root length; RDA, root diameter; TBI, total biomass.

Pearson's correlation analyses showed significant relationships between soil factors and fungal microbial community, as well as the plant growth parameters (Supplementary Table 1). Referring to the correlation coefficients (*R* values), we used structural equation modeling (SEM) to quantify the relative effects of different fungal inoculation treatments on the relationship between the growth of *A. adsurgens* and soil factors and fungal community (χ^2^ = 64.206, *df* = 57, *p* = 0.239, CFI = 0.945, RMSEA = 0.074; Figure 9). Inoculation treatment influenced soil pH and nitrate by acting on the abundance of soil fungal species thereby indirectly influencing the stem branching number, root diameter, and total biomass. The fungal diversity had a significant direct effect on soil available phosphorus, which then further influenced the total biomass. Moreover, the soil fungal community impacted soil nitrate by acting on soil ammonia and finally influenced the root diameter architecture. Subsequently, the change in root diameter significantly corresponded to the growth of root length and stem branching numbers, thus influencing the total biomass of host plants.

## Discussion

### Synergistic effect of combined fungal inoculation depending on fungal species

In this study, we determined the effects of single or combined inoculation of three root endophytic fungi on promoting the growth of desert plant *A. adsurgens* under a non-sterile substrate simulating natural drought conditions. Meanwhile, the parallel experiment of well-watered treatment was carried out when the fungus was inoculated alone, and the reduced plant height of *A. adsurgens* seedlings under drought conditions evidenced the effectiveness of imposed drought stress in our experiment (Supplementary Figure 5). Under the drought stress, we demonstrated the synergistic effects between different fungal combinations, which are consistent with that of He et al. (2022), who documented the increased dual inoculation effects of beneficial endophytic fungi between *Paraboeremia putaminum* and *Trichoderma viride* on the growth of medicinal plant *Astragalus mongholicus* under water stress conditions. However, not all fungal inoculations participated in plant growth promotion. Regardless of the single or combined inoculations, the effects mainly depended on different fungal species and combinations, which approved the previous reports that fungal species may be one of the factors influencing symbiotic relationships and plant performance (Wazny et al., 2018; Geisen et al., 2021). Liu et al. (2020b) found that the synergistic effect of arbuscular mycorrhizal fungi and endophytic fungi on tall fescue depended on the species of arbuscular mycorrhizal fungi. Yang et al. (2021) also showed that root endophytes represented an important role in promoting the biological process of the host relying on the type of host and the species of endophytes. Therefore, the use of suitable fungal endophytes, either singly or in combination, remains a major challenge as different fungal species may play different roles in plant symbiosis.

It is worth noting that in our study, the response of plant growth to fungal inoculation is different from that of the soil fungal community. The combined inoculation of ASM did not have positive effects on the growth of *A. adsurgens*, but most significantly promoted the diversity and abundance of soil fungal community. This suggests that combined fungal inoculation mediates the composition and ecological adaptation of the soil microbial community (Pang et al., 2020; He et al., 2022). Studies have shown that fungal inoculation may impact competition for niches in the fungal community, resulting in changes in species abundance (Wagg et al., 2015; Van Nuland and Peay, 2020). Due to the dependence of endophytes on plant habitat and nutrition, host plants may face tradeoffs in resource allocation among symbionts (Niwa et al., 2018). Under drought conditions, plants may allow more fungal species to coexist by controlling the abundance of dominant taxa; it may also increase or decrease certain microbial populations to alter the rhizospheric microbial community composition (Achouak et al., 2019). However, the high diversity of fungal species also means that more plant resources are needed to sustain microbial growth and reproduction, as well as more complex interspecific interactions (Voges et al., 2019; Wagg et al., 2019). Thus, the allocation of plant resources to microorganisms may be responsible for our failure to observe the fungal promoting plant growth under the combined inoculation of ASM. Additionally, the interactions between fungal species may influence plant performance by altering the way host nutrient acquisition, uptake, and metabolism (He et al., 2017; Liu et al., 2020a). Therefore, various interactions of different fungal species in plant symbiosis and competition may have important implications for host plant growth and health.

### Fungal inoculation-mediated morphological growth of *A. adsurgens* plants

Using biomass quantification as an indicator of fungal inoculation performance, we found that fungi mainly promoted the root biomass, but did not influence the shoot biomass, which may be related to the fungi-mediated morphological growth of roots (Li et al., 2019a; Jabborova et al., 2021; Toppo et al., 2022). In this study, inoculation of all fungi alone decreased the plant height of *A. adsurgens* seedlings, but significantly increased the total root length and root surface area. This may be related to endophytic fungal symbiosis cost or resource allocation. Wäli et al. (2006) have documented that seedlings of *Festuca ovina* containing *Epichloë* endophytes grew smaller than those without endophytes. Alternatively, the theory of the optimal allocation of plant resources states that plants allocate biomass preferentially to obtain the most growth-limiting resources (Puglielli et al., 2021). Thus, plants tend to invest in root growth at the expense of reduced aboveground growth in an arid environment, resulting in a decrease in aboveground plant height and biomass (Schall et al., 2012; Azizi et al., 2021). However, the decreased root diameter indicated that the fungal symbionts promote the growth and development of fine roots of *A. adsurgens* seedlings. A well-developed root system and architecture are beneficial for desert plants to improve water and nutrient absorption in deep dry soil (Likar and Regvar, 2013; Li et al., 2019a). This contributes to the ecological adaptation and resistance of host plants to adverse environments in desert habitats.

### Effects of fungal inoculation on soil properties and rhizospheric fungal community

Soil fungi interact in complex ways and play important roles in soil nutrient cycling, transformation, and promotion of plant nutrient uptake (Yadav et al., 2015; Koshila Ravi et al., 2019). In our study, the decreased soil pH under inoculations of *A. chlamydospora* may be related to the enrichment of the acidophilic fungus *Pluteaceae*. The members of *Pluteaceae* have been reported to mainly adapted to grow in acidic soil with pH 4.5–4.99 with a strong ability to decompose organic matter, thus playing an important role in soil bioremediation (Mohammadi-Sichani et al., 2017; Kunca and Pavlik, 2019). However, the increase of soil organic carbon, especially under *Monosporascus* sp. inoculation, may be related to the carbon conversion process mediated by the increased abundance of soil fungi (Kohout et al., 2018). Alternatively, the dominant group *Sordariomycetes* may also be associated with the increased organic carbon, which has been reported to favor the mineralization of soil aggregate organic matter, thereby promoting the content of soil organic matter (Xu et al., 2021). Nevertheless, the reduced soil ammonia under fungal inoculations probably corresponded to the nitrogen fixation by rhizobia. In our experiment, due to the identity of the leguminous plant, nodules were indeed observed in the roots of *A. adsurgens* plants, especially under the fungal inoculations. Therefore, the presence of adequate nitrogen-fixing bacteria may reduce the dependence of plants on microbial nitrogen mineralization (Kakraliya et al., 2018). Meanwhile, the symbiosis of nitrogen-fixing bacteria may also be related to plant growth promotion, which in turn triggers the higher demands of the host plant for other nutrients, especially phosphorus demand in N-fixing legumes (Png et al., 2017). In our study, the increases in soil available P under fungal combined inoculation were indeed observed, which was inferred to be relevant to the secretion of various hydrolases by fungi to promote the transformation of insoluble phosphorus (Fabiańska et al., 2019). Thus, we speculate that symbiotic nitrogen-fixing bacteria and endophytic fungi may exhibit complementary benefits in terms of nutrient access to their common host (Tang et al., 2019; Primieri et al., 2021). Such tri-partite symbiosis of plant-endophytic fungi-N fixing bacteria should be further explored in future studies.

In the current study, the enrichment of soil fungal communities was different under the combined inoculation treatments. Despite the acidified soil pH, inoculations of AM and AS mitigated the loss of fungal diversity and enrichment of acidophilic fungi caused by acid soils. In contrast, members of *Acremonium, Entoloma cremeoalbum, Scolecobasidium*, and *Diatrypaceae* were enriched under AS inoculation; while *Scleroramularia* and *Gaeumannomyces* were enriched under AM inoculation. *Acremonium* and *Diatrypaceae* have been widely reported to be isolated from desert halophytes and showed the ability to improve host resistance to biotic stress (Jalili et al., 2020; Ameen et al., 2021). However, *Scleroramularia* and *Gaeumannomyces* have been reported as pathogens of crops (Coombs et al., 2004; Li et al., 2011), which may be related to the transformation of endophytic fungal lifestyle. Studies have shown that the phase of endosymbiosis represents a balanced interaction between fungal virulence and host defense factors (Kuo et al., 2015). In the presence of appropriate environmental factors, saprophytic and pathogenic bacteria may transform into endophytes. Nevertheless, some beneficial fungal species, such as *Aspergillus, Psilocybe*, and *Strophariaceae* taxa, were enriched under the inoculation of ASM. *Aspergillus* has been reported to be the most common genus of desert medicinal plants and can stimulate the growth of desert medicinal plants (Ntemafack et al., 2021). *Psilocybe* was considered to be an important biological source for medical compounds and displays important research value in phytochemistry, antioxidant and cellular immunity (Nkadimeng et al., 2020). *Strophariaceae* were found as basidiomycetes, and their mycelia biomass had a significant effect on garlic scale and cucumber seed germination and seedling growth (Regeda et al., 2021). We speculate that the combined inoculation of the three fungi in this study may have promoted the increase of *Basidiomycetes* in the rhizospheric soil of *A. adsurgens*. This may also suggest that when inoculated with ASM, plants may reduce resource allocation for growth in order to recruit more beneficial microorganisms (Vandenkoornhuyse et al., 2015). According to previous studies, due to the different growth rates of fungal species, the symbiotic effects of slow-growing fungi may be delayed (Getachew et al., 2019; Vrabl et al., 2019). Consequently, considering the short duration of our pot experiment, the beneficial effects of the rhizospheric fungal microbes recruited by ASM mixed inoculation on plant growth will likely take a longer time to manifest. Moreover, the most enriched number of differential fungal species appeared in the inoculation of SM further indicating that fungal cross-inoculation plays a dominant role in the process of plant–soil ecological adaptation by adjusting the abundance and quantity of soil fungal taxa. The synergistic effect indicated that fungal combination inoculation can improve the rhizospheric ecological environment in natural habitat or the presence of a local microbiome, which is more than that of single strain inoculation (Vorholt et al., 2017).

### Interactions among plants growth, soil nutrients, and fungal community

In natural ecosystems, endophytic fungi have complex interactions with host plants and are closely related to rhizospheric microorganisms (Trivedi et al., 2020). In our study, we found that fungal inoculation mainly influenced soil nutrients by changing soil fungal species diversity and richness, thereby indirectly impacting the number of stem branches, root diameter, and total biomass of *A. adsurgens* seedlings. Certain fungal species, such as *Sordariomycetes, Chaetomium piluliferum*, and *Pleosporales*, were directly linked to soil nutrient cycling. However, in addition to the indirect effects, the fungal community themselves could also directly influence plant growth. For example, *Aspergillus, Humicola nigrescens, Pleosporaceae*, and *Lasiosphaeriaceae* were highly correlated with root growth of *A. adsurgens*. This may be due to the formation of a stable symbiotic relationship between root fungi and host plants (Bouasria et al., 2012; Yeh et al., 2019). Plants can recruit target microbial communities through signaling molecules, and subsequently exert selective pressure through the immune system, specific nutrient supply or habitat type, etc., allowing the successful colonization of beneficial microorganisms (Foster et al., 2017; Martin et al., 2017; Cordovez et al., 2019). The interaction or synergistic effect among root endophytic fungi responds to plant growth by creating favorable microflora in the rhizospheric environment of *A. adsurgens* plants. Therefore, the enrichment of beneficial microbial communities in the rhizospheric soil is crucial for plant survival and development and can confer abiotic and biotic tolerance in plants to improve fitness.

### Potential application of fungal assemblages in desert area

Promoting better growth of desert plants, improving the soil environment, and the maintenance of soil microbial diversity, are critical for the stability of arid ecosystems. In the present study, the enhanced stem branching and root growth *A. adsurgens* under *S. kiliense* and SM inoculations will help desert plants to grow better and adapt to the desert environment to promote wind prevention and sand fixation (Li et al., 2018; Zuo et al., 2020). However, soil acidification under *A. chlamydospora* and co-inoculations of AM and AS indicates that this species may be potentially applied to neutralize soil pH in alkaline areas. In contrast, *Monosporascus* sp. alone and its combined inoculation of AM and SM markedly promoted the accumulation of soil organic carbon, which is speculated to be important for the nutrient and fertility health of desert soil (Hammad et al., 2020). Most importantly, the synergistic effects of combined inoculation on the increased diversity and abundance of rhizospheric fungal community suggest that more fungal species than fewer might be conducive to regulating the composition and structure of microbial community (Mawarda et al., 2020; Cheng et al., 2021). This may be beneficial to the subsequent restoration of soil microbial functions in the desert. However, this needs to be interpreted with caution, as fungal communities do not independently in line with the plant growth-promoting effects. In desert habitats, the microbial community still includes bacteria, archaea, nematodes, and other groups, which may have a very distinct response to the endophytic fungi inoculation (Trivedi et al., 2020). Therefore, the changes in the entire microbial community may be in line with the plant growth-promoting effects and fully reflect the soil microbial functions. Based on our experimental results, inoculations of *S. kiliense* alone and SM were recommended for use in the desert plant *A. adsurgens* due to their promoted plant performance, improved soil fungal diversity, and soil organic carbon without the acidification of soil pH. Our research hints at the combined consortium of multiple fungi as a possible solution for promoting sustainable desert conservation and restoration. However, the optimal combination of strains and the complementary effects between different species should be emphasized when constructing a synthetic community (Li et al., 2021).

## Conclusion

This study explored the synergistic effects of three extremely arid habitat-adapted root endophytes, on the growth of non-host desert plant *A. adsurgens*, under drought and non-sterile conditions. Fungal inoculation markedly improved the root growth and biomass of *A. adsurgens*, and this favorable effect was dependent on specific fungal species and their combinations. However, the synergistic effect of fungal inoculation on the rhizospheric soil fungi community was inconsistent with that of plant growth. The three species combined inoculation that promoted the diversity of rhizospheric fungal community most had no effect on plant root growth when compared to the control treatments. This insinuates the differed responses between plant growth and rhizospheric fungal community to the multiple fungal interactions. Thus, the fungal species and the combined consortium used should be carefully evaluated owing to their differential effects on plant growth and soil microhabitat, as well as their complementary functional roles. Further investigation of interactions between host plants and rhizospheric soil microhabitat mediated by fungal combined inoculations are still needed to be elucidated.

## Data availability statement

The datasets presented in this study can be found in online repositories. The raw data sequences were deposited in the NCBI Sequence Read Archive (SRA) under the Bioproject number PRJNA798873 and Bio Sample accession numbers SAMN25131707 to SAMN25131730.

## Author contributions

Y-LZ and X-LH conceived and designed the experiments and wrote the manuscript. Y-LZ and Q-NH performed the experiments. Y-LZ, LQ, and J-QL analyzed the data. All authors contributed to the article and approved the submitted version.

## Funding

This study was financially supported by the National Natural Science Foundation of China (Project No. 31770561).

## Conflict of interest

The authors declare that the research was conducted in the absence of any commercial or financial relationships that could be construed as a potential conflict of interest.

## Publisher's note

All claims expressed in this article are solely those of the authors and do not necessarily represent those of their affiliated organizations, or those of the publisher, the editors and the reviewers. Any product that may be evaluated in this article, or claim that may be made by its manufacturer, is not guaranteed or endorsed by the publisher.

## Supplementary material

Supplementary Figure 1 Maximum likelihood tree based on the rDNA internal transcribed spacer (ITS) region sequences of three root endophytes isolated from extremely arid desert habitats. Sequences that were determined in the course of this study appear in bold.

Supplementary Figure 2 Rarefaction curves for the observed soil fungal operational taxonomic units (OTUs). C, non-inoculated control; A, inoculation with *Alternaria chlamydospora*; S, inoculation with *Sarocladium kiliense*; M, inoculation with *Monosporascus* sp.; AS, co-inoculation of *Alternaria chlamydospora* and *Sarocladium kiliense*; AM, co-inoculation of *Alternaria chlamydospora* and *Monosporascus* sp.; SM, co-inoculation of *Sarocladium kiliense* and *Monosporascus* sp.; ASM, combination inoculation of the three species.

Supplementary Figure 3 Non-metric multidimensional scaling (NMDS) ordination of soil fungal community composition of *A. adsurgens*. The dissimilarities of fungi were based on the Bray–Curtis method and the non-parametric ANOSIM test was used to examine the significant difference based on 999 permutations. Ellipses in the plots represent the grouping interval of fungi in different inoculation treatments. C, non-inoculated control; A, inoculation with *Alternaria chlamydospora*; S, inoculation with *Sarocladium kiliense*; M, inoculation with *Monosporascus* sp.; AS, co-inoculation of *Alternaria chlamydospora* and *Sarocladium kiliense*; AM, co-inoculation of *Alternaria chlamydospora* and *Monosporascus* sp.; SM, co-inoculation of *Sarocladium kiliense* and *Monosporascus* sp.; ASM, combination inoculation of the three species.

Supplementary Figure 4 Clustering heatmap depicting the occurrences of relatively abundant (top 50 abundance) fungal operational taxonomic units (OTUs). The color of each heat map cell indicates the relative abundance of the corresponding fungal OTUs. Cluster analysis was performed based on Bray–Curtis similarities. C, non-inoculated control; A, inoculation with *Alternaria chlamydospora*; S, inoculation with *Sarocladium kiliense*; M, inoculation with *Monosporascus* sp.; AS, co-inoculation of *Alternaria chlamydospora* and *Sarocladium kiliense*; AM, co-inoculation of *Alternaria chlamydospora* and *Monosporascus* sp.; SM, co-inoculation of *Sarocladium kiliense* and *Monosporascus* sp.; ASM, combination inoculation of the three species.

Supplementary Figure 5 Comparison of plant height under well-irrigated water and drought treatment when fungi were inoculated alone. Different lowercase letters indicate significant differences (*p* <0.05). C, non-inoculated control; A, inoculation with *Alternaria chlamydospora*; S, inoculation with *Sarocladium kiliense*; M, inoculation with *Monosporascus* sp.; WW, well-irrigated water; DS, drought stress.

Supplementary Table 1 Pearson's correlation coefficients (*R* values) between soil factors and fungal microbial community as well as the plant growth parameters.
