# Supplementary figures and images for: Species identity and combinations differ in their overall benefits to Astragalus adsurgens plants inoculated with single or multiple endophytic fungi under drought conditions
Source: Front Plant Sci. 2022 Sep 7;13:933738. doi: 10.3389/fpls.2022.933738 (PMC9490189; doi:10.3389/fpls.2022.933738)

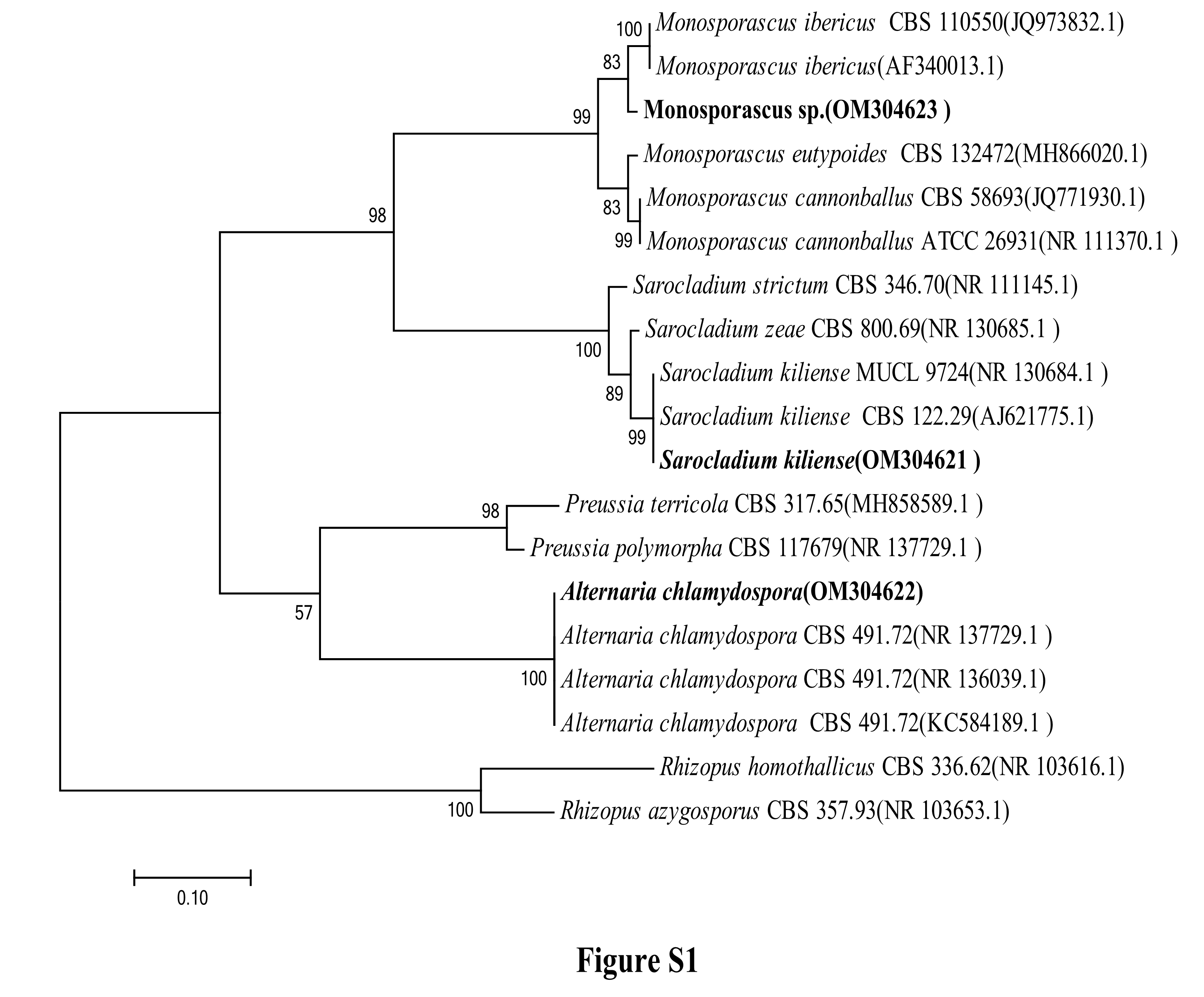

Supplement: Supplementary Figure 1 — Maximum likelihood tree based on the rDNA internal transcribed spacer (ITS) region sequences of three root endophytes isolated from extremely arid desert habitats. Sequences that were determined in the course of this study appear in bold. [file Image_1.TIF]

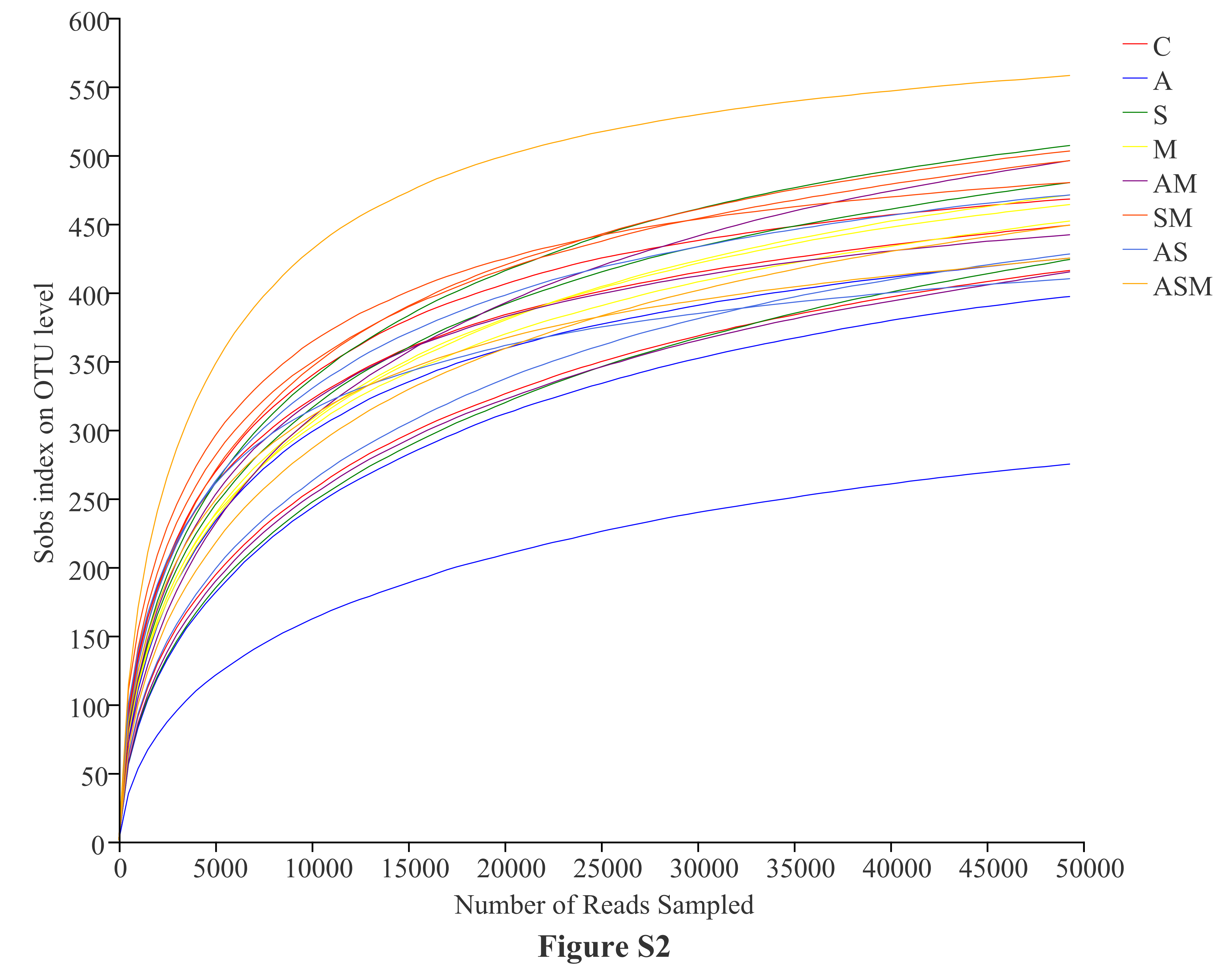

Supplement: Supplementary Figure 2 — Rarefaction curves for the observed soil fungal operational taxonomic units (OTUs). C, non-inoculated control; A, inoculation with Alternaria chlamydospora; S, inoculation with Sarocladium kiliense; M, inoculation with Monosporascus sp.; AS, co-inoculation of Alternaria chlamydospora and Sarocladium kiliense; AM, co-inoculation of Alternaria chlamydospora and Monosporascus sp.; SM, co-inoculation of Sarocladium kiliense and Monosporascus sp.; ASM, combination inoculation of the three species. [file Image_2.TIF]

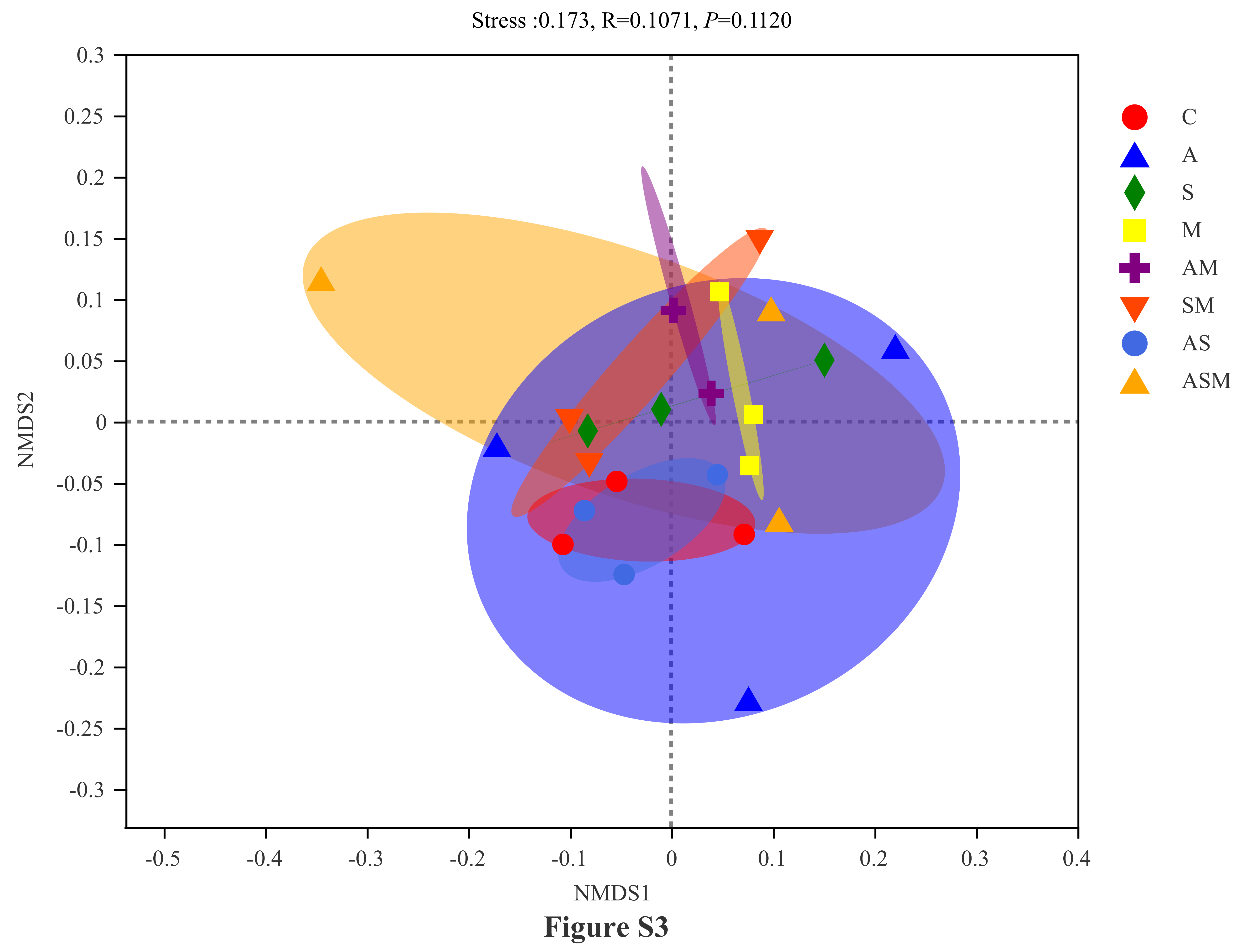

Supplement: Supplementary Figure 3 — Non-metric multidimensional scaling (NMDS) ordination of soil fungal community composition of A. adsurgens. The dissimilarities of fungi were based on the Bray–Curtis method and the non-parametric ANOSIM test was used to examine the significant difference based on 999 permutations. Ellipses in the plots represent the grouping interval of fungi in different inoculation treatments. C, non-inoculated control; A, inoculation with Alternaria chlamydospora; S, inoculation with Sarocladium kiliense; M, inoculation with Monosporascus sp.; AS, co-inoculation of Alternaria chlamydospora and Sarocladium kiliense; AM, co-inoculation of Alternaria chlamydospora and Monosporascus sp.; SM, co-inoculation of Sarocladium kiliense and Monosporascus sp.; ASM, combination inoculation of the three species. [file Image_3.TIF]

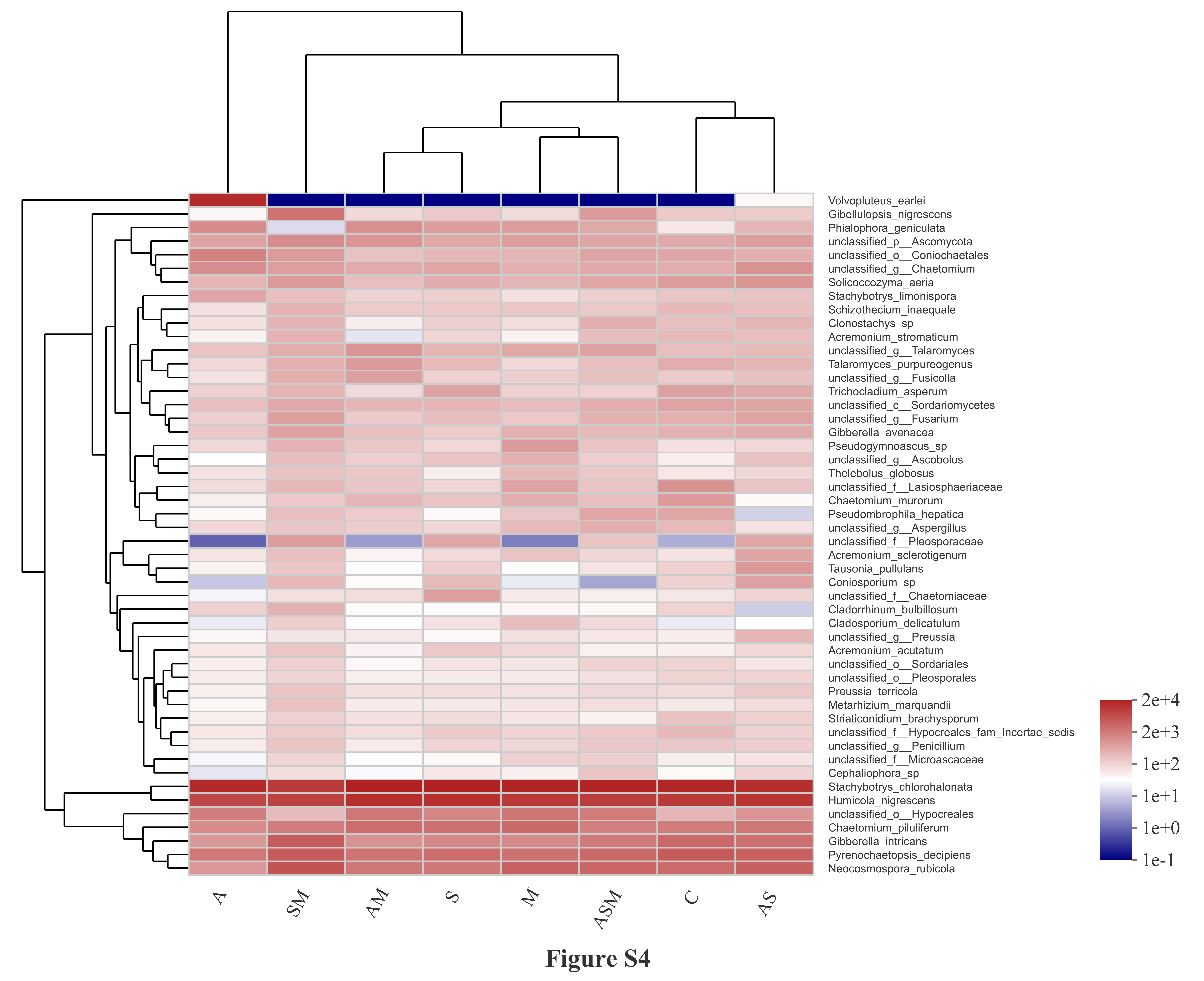

Supplement: Supplementary Figure 4 — Clustering heatmap depicting the occurrences of relatively abundant (top 50 abundance) fungal operational taxonomic units (OTUs). The color of each heat map cell indicates the relative abundance of the corresponding fungal OTUs. Cluster analysis was performed based on Bray–Curtis similarities. C, non-inoculated control; A, inoculation with Alternaria chlamydospora; S, inoculation with Sarocladium kiliense; M, inoculation with Monosporascus sp.; AS, co-inoculation of Alternaria chlamydospora and Sarocladium kiliense; AM, co-inoculation of Alternaria chlamydospora and Monosporascus sp.; SM, co-inoculation of Sarocladium kiliense and Monosporascus sp.; ASM, combination inoculation of the three species. [file Image_4.TIF]

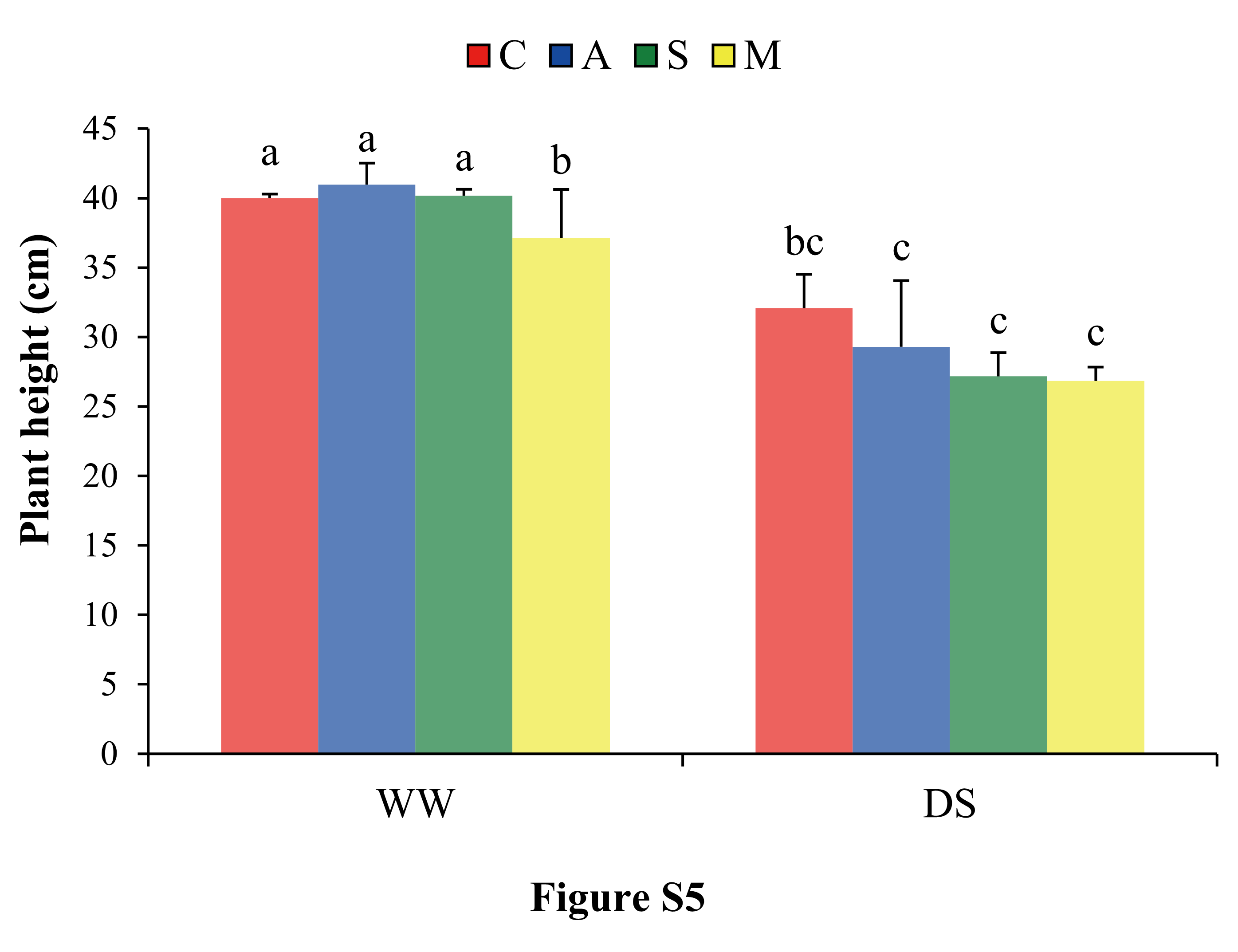

Supplement: Supplementary Figure 5 — Comparison of plant height under well-irrigated water and drought treatment when fungi were inoculated alone. Different lowercase letters indicate significant differences (p <0.05). C, non-inoculated control; A, inoculation with Alternaria chlamydospora; S, inoculation with Sarocladium kiliense; M, inoculation with Monosporascus sp.; WW, well-irrigated water; DS, drought stress. [file Image_5.TIF]
