# Supplementary material for: Species identity and combinations differ in their overall benefits to Astragalus adsurgens plants inoculated with single or multiple endophytic fungi under drought conditions
Source: Front Plant Sci. 2022 Sep 7;13:933738. doi: 10.3389/fpls.2022.933738 (PMC9490189; doi:10.3389/fpls.2022.933738)
Supplement: Supplementary Table 1 — Pearson's correlation coefficients (R values) between soil factors and fungal microbial community as well as the plant growth parameters. [file Table_1.DOC]

**Table S1** Pearson’s correlation coefficients (R values) between soil factors and fungal microbial community as well as the plant growth parameters

|  | pH | U | ACP | P | SOC | NO3 | NH4 |
| --- | --- | --- | --- | --- | --- | --- | --- |
| Fungal rhiness | 0.363 | 0.181 | 0.005 | 0.207 | -0.093 | **-0.446*** | -0.105 |
| Fungal diversity | 0.222 | 0.178 | 0.109 | **0.421*** | -0.124 | -0.367 | -0.011 |
| Simpson | -0.193 | -0.091 | -0.019 | -0.363 | -0.022 | **0.554**** | 0.105 |
| Shannoneven | 0.204 | 0.170 | 0.112 | **0.431*** | -0.111 | -0.366 | -0.010 |
| Fungal community | 0.026 | -0.262 | 0.016 | -0.189 | **0.532**** | -0.180 | **-0.441*** |
| HEI | -0.027 | 0.355 | **0.448*** | 0.391 | -0.162 | -0.119 | 0.341 |
| STB | **-0.501*** | 0.088 | -0.341 | **0.430*** | 0.093 | -0.291 | -0.131 |
| RLE | -0.034 | -0.308 | -0.054 | -0.159 | 0.061 | -0.207 | -0.048 |
| RSA | -0.047 | -0.247 | 0.060 | -0.170 | -0.102 | -0.005 | 0.032 |
| RDA | 0.262 | 0.083 | 0.342 | -0.098 | -0.070 | **0.499*** | 0.228 |
| IAA | -0.063 | **0.424*** | **0.434*** | 0.357 | -0.105 | 0.192 | 0.053 |
| SBI | -0.399 | -0.012 | 0.034 | 0.318 | 0.013 | -0.082 | 0.070 |
| RBI | -0.215 | -0.339 | -0.082 | -0.013 | 0.101 | -0.095 | -0.006 |
| TBI | -0.360 | -0.068 | 0.034 | 0.261 | 0.017 | -0.090 | 0.053 |
| TN | 0.007 | **-.405*** | -0.308 | -0.316 | 0.374 | -0.124 | 0.171 |
| TP | -0.341 | **-.550**** | -0.275 | -0.092 | 0.254 | -0.083 | -0.207 |

U, soil urease; ACP, soil acid phosphatase; P, soil available phosphorus; SOC, soil organic carbon; NO3, soil nitrate; NH4, soil ammonia; HEI, plant height; STB, stem branching numbers; RLE, root length; RSA, root surface area; RDA, root diameter; IAA, indole acetic acid; SBI, shoot biomass; RBI, root biomass; TBI, total biomass; TN, plant total nitrogen; TP, plant total phosphorus. **P* < 0.05; ***P* < 0.01.
